# The Symbiosis Interactome: a computational approach reveals novel components, functional interactions and modules in *Sinorhizobium meliloti*

**DOI:** 10.1186/1752-0509-3-63

**Published:** 2009-06-16

**Authors:** Ignacio Rodriguez-Llorente, Miguel A Caviedes, Mohammed Dary, Antonio J Palomares, Francisco M Cánovas, José M Peregrín-Alvarez

**Affiliations:** 1 Dpt. of Microbiology and Parasitology, University of Seville, Seville, Spain; 2 Dpt. of Molecular Biology and Biochemistry, University of Malaga, Malaga, Spain; 3 Molecular Structure and Function, SickKids Research Institute, 555 University Avenue, Toronto ON, M5G 1X8 Canada

## Abstract

**Background:**

*Rhizobium*-Legume symbiosis is an attractive biological process that has been studied for decades because of its importance in agriculture. However, this system has undergone extensive study and although many of the major factors underpinning the process have been discovered using traditional methods, much remains to be discovered.

**Results:**

Here we present an analysis of the 'Symbiosis Interactome' using novel computational methods in order to address the complex dynamic interactions between proteins involved in the symbiosis of the model bacteria *Sinorhizobium meliloti *with its plant hosts. Our study constitutes the first large-scale analysis attempting to reconstruct this complex biological process, and to identify novel proteins involved in establishing symbiosis. We identified 263 novel proteins potentially associated with the Symbiosis Interactome. The topology of the Symbiosis Interactome was used to guide experimental techniques attempting to validate novel proteins involved in different stages of symbiosis. The contribution of a set of novel proteins was tested analyzing the symbiotic properties of several *S. meliloti *mutants. We found mutants with altered symbiotic phenotypes suggesting novel proteins that provide key complementary roles for symbiosis.

**Conclusion:**

Our 'systems-based model' represents a novel framework for studying host-microbe interactions, provides a theoretical basis for further experimental validations, and can also be applied to the study of other complex processes such as diseases.

## Background

Plant-microbe interactions play an important role in agriculture and a lot of effort has been dedicated to analyse these interactions in detail. One of these interactions is the *Rhizobium*-Legume symbiosis, a process that allows the growth of the plant in the absence of externally supplied nitrogen. This is a well studied agronomically important process that is also used as a model to study general genetic aspects of plant-microbe interactions [1,2]. Rhizobial bacteria and legumes have evolved complex signal exchange mechanisms in which a lot of genes are involved [3]. To probe this complexity further we chose to study the model rhizobial symbiont genome *Sinorhizobium meliloti *[4]. *S. meliloti *is a model bacterium that can engage in a symbiotic interaction by infecting the roots of members of the genera *Medicago *and *Melilotus*, being the *S. meliloti-Medicago truncatula *interaction the model system for indeterminate type nodules [5].

The sequencing of hundreds of complete genomes from diverse species is having a tremendous impact on our understanding of biology by enabling the identification of all proteins and the analysis of their function. Despite the vast body of literature about the *Rhizobium*-legume interaction there have been no systematic large-scale attempts to identify its components and function using a systems biology perspective, and most studies have been restricted to the analysis of individual proteins. However, biological functions results from the interactions of proteins so that understanding the network of biological linkages utilizing functional genomics information is becoming a hot topic in current research projects [6-11]. The main advantage of creating these networks lies in the ability to understand biological processes from a system level perspective. This would ideally require the application of computational and experimental techniques to combine experimental observations of protein-protein interactions (PPIs) and computational predictions derived from different data sources. To date a variety of methods have been developed to derive large scale networks of PPIs for a variety of organisms. These range from experimental methods such as yeast two-hybrid screens, or tandem affinity purification coupled with mass spectrometry [6,8,9,12], to computational methods such as genome context methods [13,14]. The integration of these types of data helps to provide a complete overview of gene networks of high value for characterizing many biological processes, and ultimately, for understanding the basis of host-microbe interactions including diseases [15-17]. However, experimental information is sometimes missing and deriving gene networks from different computational approaches is not an easy task. Computational predictions such as those obtained by applying genome context methods usually measure functional interactions between proteins. The assumption is that proteins are most likely to interact if: a) their proteins are either present or absent together across multiple genomes (the Phylogenetic Profile method) [18]; b) a gene fusion event occurred in other species (the Gene Fusion or Rosetta Stone method) [19,20]; c) the genes are in physical proximity (the Gene Cluster method) [17]; or d) the genes are conserved in physical proximity and in phylogenetically distant genomes (the Gene Neighbor method) [21]. These methods have the advantage over experimental methods and other computational methods based on protein conservation such as Interologs [22] or literature mining [23], that they are not biased towards well studied or conserved proteins or interactions [24]. Therefore, genome context methods are able to highlight organism-specific features since they just rely on genome structure. The outputs derived by these methods can be computationally integrated in order to reconstruct network models of the relations between genes [13,14]. Data integration for inferring protein associations is advantageous for two main reasons. First, combining data from diverse studies and methods generates data sets of higher quality, and second, integration effectively captures different aspects of organism's biology [25-27]. Further exploiting the topological properties of these networks, clustering algorithms have subsequently allowed proteins to be organized into discrete interconnected units known as functional modules representing either protein complexes or biochemical pathways [28,29]. In addition, integration of additional functional and comparative genomics data sets are further providing insights into how these modules and their components are co-ordinated and how they may have evolved [9,30].

Due to the scarcity of large-scale experimental assays aiming to study this important microorganism-host interaction, we chose to apply a systems-based computational approach to evaluate and organize our current knowledge about this complex biological process further. Here, we first reconstruct an extensive and accurate functional network in *S. meliloti *by integrating the functional associations present in the two well known databases PROLINKS [13] and STRING [14] (see methods). These databases host functional linkage predictions obtained mainly by the four different computational genome context approaches described above. Second, we present an analysis of the 'Symbiosis Interactome' (a detailed functional interaction network of the proteins involved in the *S. meliloti*-Legume symbiosis) by first mapping proteins known to be involved in symbiosis on top of the *S. meliloti *network, and secondly, by extending this resulting network by means of a novel method, referred here as 'phenotypic profiling', which is further extended by incorporating data from the computational prediction of functional modules. This computational approach potentially revealed the complex interplay of functional interactions between proteins involved in *S. meliloti-Medicago *symbiosis providing a way to expand the current understanding of symbiosis by enabling hypothesis generation based on our predicted network. Finally, since one of the major advantages of constructing PPI networks is the ability to predict functions for proteins based on their association with well known proteins, we identified and tested the functions of candidate proteins and demonstrate that novel Symbiosis Interactome proteins can still be discovered despite the many decades of effort dedicated to study this important and complex biological process.

## Results

### The *S. meliloti *network

**Figure 1:**
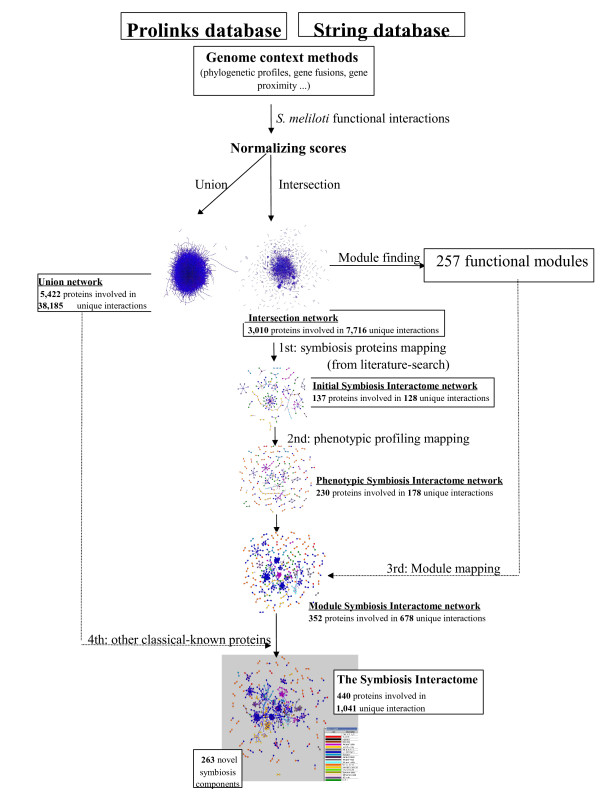
**Generation of the Symbiosis Interactome network**. Schematic overview of the generation of the *S. meliloti *functional network, prediction of functional modules, and generation of the Symbiosis Interactome network.

An initial template functional network was generated from the confident interactions obtained by merging *S. meliloti *functional genomics data hosted by the PROLINKS [13] and STRING [14] databases (see methods) (see Fig. 1 for a schematic and full description of the approach). These data were derived by using the following genome context methods: the Phylogenetic Profile method [18], which uses the presence and absence of proteins across multiple genomes; the Gene Cluster method [17], which uses genome proximity; Rosetta Stone [19,20], which uses a gene fusion event in a second organism; and the Gene Neighbor method [21], which uses both gene proximity and phylogenetic distribution. In this model, some linkages may represent direct physical protein-protein interactions (PPIs) and other are functional associations (not mediated by physical contact) such as regulatory, genetic or metabolic associations. For the purpose of this study, we call these linkages functional interactions. Therefore, our integrated network represents a description of functional coupling between genes in *S. meliloti*. We combined and integrated these data into two non-redundant datasets formed by the interactions present in both databases (the intersection network), consisting of 3,010 proteins (48% of the *S. meliloti *proteome) involved in 7,716 functional interactions, and the union of both data sets (the union network), consisting of 5,422 proteins (87% of the *S. meliloti *proteome) involved in 38,185 functional interactions. The original confidence scores of the interactions present in the two databases were integrated and re-scored, and the resulting networks were validated by calculating the Area Under ROC Curves (AUC) [see Additional file 1]. The analysis showed that the intersection dataset has a higher accuracy (AUC = 0.75) than the union dataset (AUC = 0.69), and these two networks have bigger accuracies than any of the two independent databases. Based on the results, unless otherwise noted, the intersection *S. meliloti *network was chosen for further analyses.

**Figure 2:**
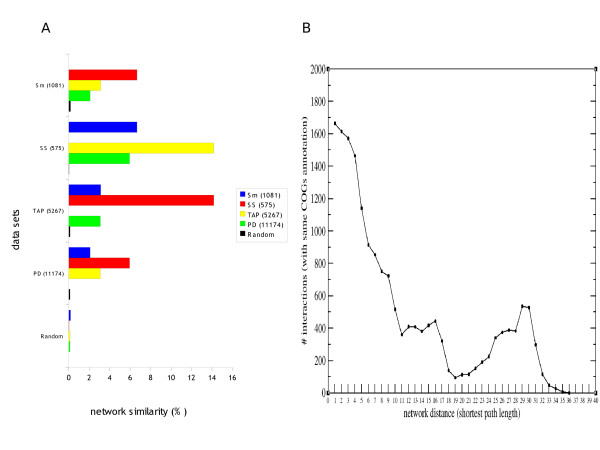
**Network analyses**. (A) Overlap of the *S. meliloti *network with three experimentally derived networks and randomly generated networks. Sm stands for *S. meliloti *network. SS and TAP refer to small- and large-scale experimental networks, respectively, present in DIP [31]. PD refers to a recently published pull down study [7]. Random refers to the average values of 100 random networks created with the same number of nodes and interactions as the Sm dataset. Error bars are negligible and not shown for clarity. (B) COGs [37] annotation from functional interactions and its correlation with network distance. 'X' axis stands for different network distances (shortest path lengths) in the *S. meliloti *network. Values at the 'Y' axis stand for the number of interactions having the same COGs categories in both interacting partners.

Unfortunately, the lack of large-scale protein interaction data on *S. meliloti *make it impossible to validate experimentally this initial functional network. However, besides calculating AUCs, we can also assess the quality of our preliminary intersection network by comparison with other available bacterial interaction datasets. We thus compared our predicted network with three other *Escherichia coli *experimental interaction data sets: one small- and one large-scale datasets obtained from the Database of Interacting Proteins (DIP) [31], and a third large-scale dataset recently published [7]. Reciprocal best-hits *S. meliloti *orthologs of *E. coli *were used to predict *S. meliloti *interologs (see methods). The network similarity (NS) between our network and the small-scale *E. coli *data was 6.6%, significantly more than random networks (0.03%(aver) (p < 0.001) (Fig. 2a). The overlap between our network and the large-scale *E. coli *data was also significantly greater than expected by chance. Furthermore, the NS of *S. meliloti *interactions versus small-scale is higher than versus large-scale assays, and the overlap with the small-scale data is similar to that for the *E. coli *large-scale data sets. The number of proteins shared between each other data sets was also comparable (data not shown). The results show that the predicted network also has similar rates of true positives, false positive and negatives compared to other high-quality experimental networks further demonstrating its accuracy, and potential for hypothesis-generation and further experimental validation. Nonetheless, for other type of analyses, such as accuracy prediction using only computational methods as reference datasets or network evolution studies, it might be more relevant to compare functional linkage data from two sources (for example, *S. meliloti *versus *E. coli*), rather than functional linkages of one organism and physical interaction of another.

The *S. meliloti *network demonstrated to have properties of scale-free network [see Additional file 1] like other biological networks, the Internet and social networks [32]. Most of the proteins had few interacting partners, where a subset of 'hubs' form a far greater number of connections. Scale-free networks are predicted to be robust against random node removal but vulnerable to hub removal, a property that might be preserved across evolution [32]. Furthermore, the average clustering coefficient (ACC) of the intersection network and its diameter or average shortest path length (L) (see methods) suggests properties of a small-word network (L ~ Lrandom, ACC >> ACCrandom) typical of intracellular network in which the nodes are connected when they are involved in the same biological processes [32].

### Prediction of functional modules

While defining accurate PPI networks is important, the ultimate goal of interactome analyses is to identify the functional modules in these networks, that is, proteins with related functions that tend to be clustered into highly interconnected subnetworks [10,33,34], and to validate them. To assess if our network could also be clustered into such subnetworks, we first tested the capacity of the *S. meliloti *network to form groups of highly interconnected proteins, as indicated by its Average Clustering Coefficient (ACC) (see methods). Indeed, the ACC of the *S. meliloti *network is much higher (ACC = 0.41) than other large-scale *E. coli *(ACC = 0.15 [31] and ACC = 0.08 [7]) and *H. pylori *[6] (ACC = 0.02) experimental, and random networks (ACC = 0.0002) suggesting the organization of the *S. meliloti *network in functional modules.

**Figure 3:**
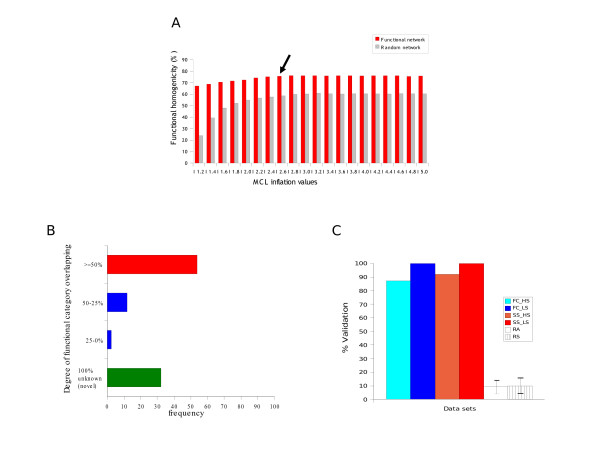
**Functional module analyses**. (A) Bars represent the average percentage of overlap in COGs [37] categories within module predictions at different MCL inflation values [35] for the *S. meliloti *(Sm) network and the average values of 100 random networks created with the same number of nodes and interactions as the Sm dataset. Black arrow stands for the Inflation value chosen for further analyses. Error bars are negligible and not shown for clarity. The percentage of overlap for each module was obtained by considering only the most abundant COGs category in the module. (B) Functional overlap of modules related to membership of COGs functional categories. (C) Accuracy of the functional network to predict correct COGs (FC) and symbiosis-stage annotations (SS) [see Additional file 2] in functional modules using a leave-one-out cross-validation procedure. Bars indicate the frequency of correct COGs/symbiosis-stage assignments. Two measures of stringency were employed: high (HS) and low stringency (LS). RA and RS refers to random networks of equal size to the *S. meliloti *network generated for COGs and symbiosis-stage correct annotation comparisons, respectively. Error bars indicate standard deviation for 100 replicate random controls.

We further predicted the structure of these subnetworks by using the Markov Cluster (MCL) algorithm [35] (see methods). MCL simulates random walks within graphs using the language of Markov (stochastic) matrices in order to partition a graph into highly connected clusters. This procedure works efficiently on large dense graphs [9], and have the advantage over other methods such as PathBlast [36] that does not rely on conservation, therefore, being able to detect species-specific clusters. Nonetheless, the performance of different clustering methods varies widely and usually drops for networks including noisy data. Reliable criteria for evaluating the quality of the predicted modules are also lacking, making difficult to compare the results obtained by different clustering procedures, or to assess the biological relevance of the predicted modules. In our study we chose MCL because of our own experience and expertise and the fact that it has been applied to numerous key studies, showing MCL clustering as one of the state of the art methods for network clustering [[9,29], Peregrín-Alvarez JM, Xiong X, Su C, Parkinson J: The modular organization of protein interactions in *Escherichia coli*, submitted]. The network was thus organized into 345 highly interconnected clusters containing three or more proteins [see Additional file 2]. Modules derived from our *S. meliloti *network appear to be much more functionally homogeneous (Fig. 3a) and produced similar distributions of module sizes (data not shown) compared with the modules derived from random networks highlighting the non-random organization of proteins into modules. 185 (54%) of these predicted clusters possessed a high proportion (>= 50%) of common Clusters of Orthologous Groups (COGs) functional annotations [37] (Fig. 3b). These results *a priori *suggest that most of these predicted clusters correspond to known functional modules in the form of protein complexes, metabolic or regulatory pathways. Less functionally well defined modules (14%) may correspond to multi-functional modules involved in pathway cross-talk (component annotations are heterogeneous). The remaining may represent novel functional modules (component annotations are absent) (see below). To compute the significant of finding specific COGs functional modules, we generated 10,000 random module sets of the same size, and counted the number of times we found each COGs module in each randomized network. For each module, a p-value was computed based on the distribution of the random sets (assuming a normal distribution) and our module predictions, therefore, representing the probability of seeing such modules at chance. This yielded 121 clusters (58%, out of 209 that we were able to compute statistics) that were significantly enriched in COGs functional categories (p < 0.01) [see Additional file 2], thus likely representing true functional modules. An additional 136 clusters had no COGs assignments (i.e component annotations are absent) thus potentially representing novel functional modules (Fig. 3b and Additional file 2) (see methods). Finally, a total of 257 modules (121 statistically significant and 136 novel) were considered for further network analyses (see below).

### The Symbiosis Interactome network

We first undertook an exhaustive literature-search analysis to identify and compile a list of bacterial proteins whose role in the symbiosis *Rhizobium*-Legume has been widely studied (Additional file 2 and methods). These proteins were classified as "classical-known" proteins in different categories according to the stage of symbiosis they are involved in.

**Figure 4:**
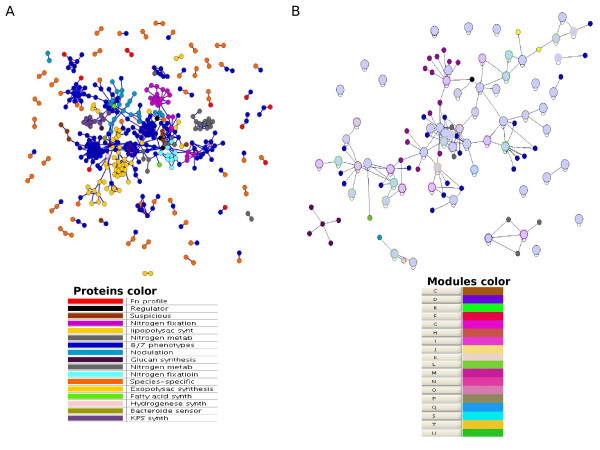
**The Symbiosis Interactome network and its organization into functional modules**. (A) Network visualization of the 440 proteins (nodes) and 1,041 interactions (edges) potentially involved in the *S. meliloti*-Legume symbiosis. The colours of each protein indicate the stage of symbiosis [see Additional file 2] or the phenotypic profile categories the proteins belong to (see panel). Edges are coloured according to the probability of interaction, from blue (less probability) to red (more probability). We used Biolayout [66] for network visualization. (B) Graphical overview of the interconnected functional modules predicted over the Symbiosis Interactome network presented in (A). Larger pie charts (blue sky colour) represents individual functional modules (only modules with 3 or more proteins are shown) and smaller pie charts represents proteins not predicted to be part of functional modules and directly interacting with modules, and coloured as in (A). Module borders are coloured if >60% of their members are associated with a single COGs category (black otherwise)(see panel). We used Cytoscape [67] for network visualization. COGs codes are as follow: [J] Translation, ribosomal structure and biogenesis; [A] RNA processing and modification; [K] Transcription; [L] Replication, recombination and repair; [B] Chromatin structure and dynamics; [D] Cell cycle control, cell division, chromosome partitioning; [Y] Nuclear structure; [V] Defense mechanisms; [T] Signal transduction mechanisms; [M] Cell wall/membrane/envelope biogenesis; [N] Cell motility; [Z] Cytoskeleton; [W] Extracellular structures; [U] Intracellular trafficking, secretion, and vesicular transport; [O] Posttranslational modification, protein turnover, chaperones; [C] Energy production and conversion; [G] Carbohydrate transport and metabolism; [E] Amino acid transport and metabolism; [F] Nucleotide transport and metabolism; [H] Coenzyme transport and metabolism; [I] Lipid transport and metabolism; [P] Inorganic ion transport and metabolism; [Q] Secondary metabolites biosynthesis, transport and catabolism; [R] General function prediction only; [S] Function unknown; [-] Non-annotated genes.

To place the bacterial proteins involved in symbiosis (here referred as Symbiosis Interactome) in biological context, the 'classical-known' proteins were used to generate a subnetwork of this complex biological process (Fig. 1). A total of 92 *S. meliloti *'classical-known' components were then used to anchor a subnetwork of functional interactions within the *S. meliloti *network (see methods). This yielded our 'initial Symbiosis Interactome network' formed by 137 proteins involved in 128 interactions. From the classical-known list the only gene names that did not map any of the two functional networks (intersection and union) were the genes *nov*, *cps*, *vis *and *mos*. These mostly represent either genes not known in *S. meliloti *or absent in the networks we generated. The resulting subnetwork was further extended based on the expectation that: first, the 'classical-known' components identified by our preliminary literature curation should remain central to the network, and secondly, neighbor proteins of those classical-known components in the *S. meliloti *network are more likely to participate in similar biological processes (Fig. 2b). This is consistent with the idea that interacting proteins in the network often function in the same pathway or protein complex and, therefore, close network neighbors of the classical-known proteins may be potentially involved in symbiosis. The preliminary Symbiosis Interactome set was then extended by allowing the addition of other proteins absent from the literature-search but predicted from our *S. meliloti *map. This was done through three extra rounds of node and edge additions: first, adding first-level indirect interactions (i.e. direct interactors of our initial network), and mapping phenotype-specific information by mean of a novel method referred here as 'phenotypic profiling' (see methods). This was done by adding nodes with the following phenotypic profiles: "Fn", proteins with homologs in other plant-nitrogen fixing organisms or *S. meliloti*-specific genes to account for genes potentially involved in symbiosis; "FnFl", proteins with homologs in other nitrogen fixation free-living organisms; and "CFlFnPpPSSyO" and CfnPpPSSyO", the two most common phenotypic profiles obtained from the list of 'classical-known' proteins [see Additional file 2]. Table 3). This resulted in an extension of our initial Symbiosis Interactome map by adding both the proteins with these profiles and their linkages mapping the intersection *S. meliloti *network. We also allowed additional functional linkages formed by one component with a "Fn" or "FnFl" profile, and the other component having any of the profiles mentioned above, since we hypothesized that these new linkages may represent novel interactions involved in symbiosis. We called this network 'the phenotypic network' formed by 230 proteins involved in 178 interactions. Secondly, we mapped functional modules [9,33,34] (see methods, Fig. 3 and Additional file 2) on top of the initial Symbiosis Interactome network to account for the completion of the functional modules the classical-known and other direct-neighbors proteins may participate in. We called this 'the module network' formed by 352 proteins involved in 678 interactions. Finally, we added those 'classical-known' proteins that did not map the intersection *S. meliloti *network but did map 'the union *S. meliloti *network' (see methods) to extend the module network with the only condition that these proteins have to interact with themselves or with any of the proteins present in 'the module network'. The final 'Symbiosis Interactome network' contained a total of 440 nodes (classical-known and novel proteins) and 1,041 edges (functional interactions) (Fig. 4a and Additional file 3). Using the SIGCLEAVAGE software [38] we predicted the periplasmic location of all *S. meliloti *proteins (see methods). The accuracy of SIGCLEAVAGE has been reported to be high and similar to other computational approaches, therefore the proteins predicted as periplasmic or cytoplasmic are likely to be of correct subcellular localization. Interestingly, we found that 282 (64%) of the 440 Symbiosis Interactome components have a predicted signal peptide [see Additional file 2]. This is consistent with recent studies showing that a large number of periplasmic proteins are upregulated during symbiosis [39-43]. Our results suggest a membrane location in a high proportion of network proteins and potential involvement in plant-microorganism interactions [44]. Furthermore, our results show that the Symbiosis Interactome does not act in isolation, rather it involves many interactions formed by highly conserved proteins participating in many other well known cellular functions (Fig. 4b). In addition, like the intersection *S. meliloti *network, the ACC of the the Symbiosis Interactome and its diameter suggests properties of a small-world and scale-free topology [see Additional file 1], highlighting both the complexity of this subnetwork, and its robustness to node attacking [32].

### Prediction of functional annotation and stage of symbiosis

A major goal for many functional genomics and proteomics projects is the generation of accurate functional information for every gene and its product. Although tremendous progress has been made through the application of such systematic studies, we found that within the *S. meliloti *proteome 3,376 (54%) proteins were not assigned to a functional category according to COGs, 290 (5%) have been assigned category S (function unknown), and a further 307 (5%) proteins have only been assigned into category 'R' ('general function prediction'). There has been recent progress in the development of novel methods of functional inference based on network connectivity [45]. The availability of our *S. meliloti *functional network thus provides a valuable resource for future studies aimed at predicting the functions of these high number of functionally 'orphan' proteins. In order to test the ability of our functional network to accurately infer reliable functional annotations and the stage of symbiosis where components of the Symbiosis Interactome may participate, we investigated a basic network-based approach based on functional category membership within predicted functional modules. To provide estimates of the accuracy of functional modules on inferring reliable functional annotations, we applied a cross-validation procedure to predict functional annotations (see methods). We were able to identify correct annotations for 87%–100% of the proteins contained in modules depending on the stringency of COGs category assignments (see methods and Fig. 3c). The accuracy of this type of functional module predictions has been found to be superior to other methods based merely on direct interacting partners [Peregrín-Alvarez JM, Xiong X, Su C, Parkinson J: The modular organization of protein interactions in *Escherichia coli*, submitted]. These findings highlight both the quality of the network and the predicted functional modules for hypothesis generation and future experimental validation.

Based on these results, module 266, for example, includes three proteins *Q92QS6 *(Smc01792), *Q92QS4 *(SMc01794) and *Q92VP9 *(Smb21071) [see Additional file 2]. The first two proteins are involved in M (cell wall/membrane/envelope biogenesis) while the third one has no COGs category assignment. We therefore predict the latter is potentially involved in this biological process. Furthermore, interestingly, we correctly identify the stage of symbiosis for 92%–100% of the proteins contained in modules depending on stringency (see methods and Fig. 3c). Again based on these promising results, module 208, for example, includes two nodulation proteins: *nodP2 *(Smb21223) and *nodQ2 *(Smb21224); and the novel protein *Q92VH5 *(SMb21225) [see Additional file 2], therefore, being tempting to speculate the participation of the latter in nodulation.

### The conservation and evolution of the Symbiosis Interactome network

To investigate the conserved nature and evolution of our predicted Symbiosis Interactome network, the classical-known and novel Symbiosis Interactome components were classified into different node ages according to their phylogenetic distribution (see methods). A total of 313 (~ 68%) proteins were classified as old nodes (with broad phenotypic profiles (i.e with homologs in 7 or 8 phenotypic categories) suggesting an old evolutionary origin for symbiosis [8,46]. Furthermore, of the 92 classical-known proteins previously identified as components of the Symbiosis Interactome 62 (~ 67%) had homologs with distantly related genomes, indicating that these highly conserved proteins were a valid system from which to derive a model of symbiosis. In addition, highly conserved genes tend to involve essential genes [8,9]. Since most of the genes known to be involved in symbiosis are highly conserved [see Additional file 2] this suggests that these genes could be essential for organism's survival or at least determinant for symbiosis. Indeed, many of the novel genes predicted by our approach are missing from a *S. meliloti *mutant collection recently published [47] (data not shown) suggesting an essential role for many of these novel genes. It has also been shown that nodes with high network connectivity tend to be essential nodes [8,9,15]. Since most of the 'classical-known' and other novel Symbiosis Interactome proteins have multiple interacting partners (315 (~ 68%) and 341 (~ 74%) proteins using the Symbiosis Interactome and the complete intersection *S. meliloti *network, respectively, interact with more than one protein in the network) (see methods), this suggests that these proteins may indeed have a key role in this important biological process. It follows from these findings that the number of interactions of the Symbiosis Interactome proteins are positively correlated with its conservation [see Additional file 1] supporting a model of evolution of the Symbiosis Interactome from core components by adding additional ones over time [46].

### Experimental validation

**Figure 5:**
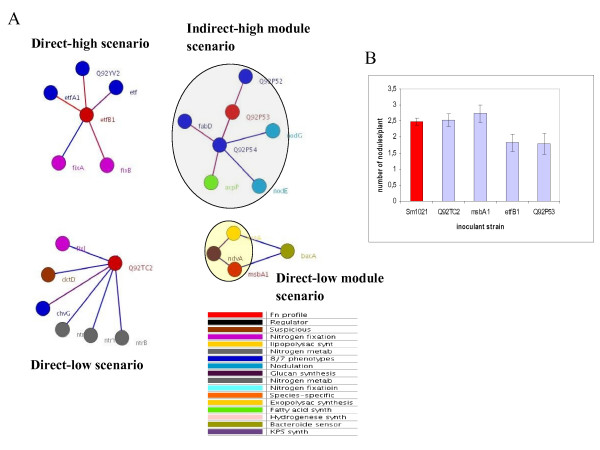
**Targeted novel proteins and experimental validation**. (A) Visualization of the four novel predicted proteins selected for experimental validation based on four different network scenarios: direct-high, direct-low, direct-low module, and indirect-high module scenarios. Only direct interactors of the targeted proteins are represented but for the last scenario that involves indirect interactions. The colours of each protein indicate the stage of symbiosis or phenotypic profile categories the proteins belong to (see panel). Large coloured ovals indicate functional modules with different colours indicating their involvement in different clusters. The colours of each protein as in Fig. 4. Edges are colored according to the probability of interaction, from blue (less probability) to red (more probability). We used Biolayout for network visualization. (B) Nodulation assays of different mutants compared to *S. meliloti *1021 wild type strain. Results are expressed as number of nodules per plant. Bars represent the average of three independent experiments (see methods). Red bar stands for control experiments, and blue bars for the four genes tested in this study.

Examination of proteins in the *S. meliloti *network revealed that proteins involved in the same biological process tend to interact directly or being in close proximity to each other (Fig. 2b). For example, 1,666 interactions (77%), out of 2,159 for which we could obtain COGs annotations for both interaction partners, have the same COGs annotation assigned to both protein partners at distance 1 (i.e. those interactions present in our *S. meliloti *network), compared to 9% (average value) of interactions with the same COGs annotation using 100 random networks, thus, providing an indirect measure of network accuracy (data not shown). The Symbiosis Interactome network presented here, therefore, can be used to predict the biological role of unknown proteins based on the functions of their interacting partners, as demonstrated for other PPI networks [8,9,34]. Therefore, a major goal of this study was to find novel components among many proteins of still unknown function in symbiosis. However, the validation of computational methods is a major issue in systems biology because only a small fraction of predictions can be tested experimentally with reasonable time and costs. Many predictions can be then summarized as 'priority lists' of potential proteins involved in a biological process or with a particular function. To demonstrate the implication of these novel symbiosis components, and, at the same time, beginning to validate our Symbiosis Interactome map and our approach, we have studied the symbiotic properties of several *S. meliloti *strains mutated in novel genes, and provide the rest of predictions as "priority list" for future experimental validation. The selection of novel genes to be experimentally tested was guided by using four different network scenarios (one targeted gene per scenario) (see Fig. 5a): direct-high scenario, novel proteins supported by direct network evidence at high probability (that is, novel genes directly interacting with classical-known symbiosis genes at high probability) by targeting protein *etfB1 *(interacts with *fixA *and *fixB*); direct-low scenario, novel genes supported by direct network evidence at low probability by choosing protein *Q92TC2 *(interacts with *dctD*, *fixJ *and *ntr *genes); direct-low module scenario, novel genes supported by direct evidence in functional modules at low probability (that is, novel genes directly interacting with classical-known genes in the same functional module) by targeting protein *msbA1 *(interacts with *bacA *and *ndvA*, and is in the same module as *ndvA *and *exsA*) [see Additional file 2]; and indirect-high module scenario, novel genes supported by indirect evidence in functional modules at high probability (that is, novel genes indirectly interacting with classical-known genes in the same functional module) by choosing protein *Q92P53 *(interacts with *Q92P52 *and *Q92P54 *which in turn interacts with *acpP*, *nodE*, *nodG *and *rkp *genes at high probability) [see Additional file 2].

*M. sativa *plants were inoculated with *S. meliloti *strains mutated at these genes, using *S. meliloti *1021 as control wild-type strain (see methods). We could not observe any difference in nodulation phenotypes between plants inoculated with the strain mutated in *Q92TC2 *and the 1021 control strain (Fig. 5b). On the other hand, differences in nodulation were observed when plants were inoculated with the other mutants. A 20–30% decrease in nodule number (depending on the experiment these are maximum and minimum values) was observed in plants inoculated with the strain mutated in *etfB1*, and a 20–25% decrease in nodule number in plants inoculated with the mutant in *Q92P53*. These differences have been shown as biologically significant in other symbiosis studies [48-50]. In addition, it is important to notice that a high percentage of small nodules (white and probably non-fixing nodules) was also observed in plants inoculated with *etfB1 *mutant. Surprisingly, plants inoculated with the strain mutated in *msbA1 *showed a 20–25% increase in nodule number when compared with control strain (Fig. 5b). In summary, these results clearly suggest that still there could be a number of non-described proteins involved in the *Rhizobium*-Legume interaction.

### Further functional predictions

Based on our experimental results and the interactions of the novel targeted proteins, *etfB1 *acts in a module involved in energy production and conversion, and we predict it to be potentially involved in nitrogen fixation [see Additional file 2]; in fact, the high percentage of small non-fixing nodules induced by the strain mutated in this gene is consistent with this role. *msbA1 *functions in a module together with *ndvA *and *exsA *genes and is potentially involved in glucan synthesis; and *Q92P53 *is functioning within a module involved in lipid transport and metabolism in coordination with *nod *genes, and may be potentially involved in the regulation of the first stages of nodule formation. These novel findings only represents hypothesis and still have to be analysed in more detail to shed more light on their precise biological role and mechanistic details but, nonetheless, the predictions highlighted here represent a tempting guide for further experimental validation.

## Discussion

The building of our final 'Symbiosis Interactome network' complemented our initial classical-known list in many different ways. First, we extended the initial set from 92 to 163 known components (92 from the intersection and 71 from the union network). Second, we identified 263 potential novel Symbiosis Interactome components, representing ideal targets for further experimental validation [see Additional file 2]. Third, the incorporation of functional modules in the network provides additional information concerning the structure and functional organization of the Symbiosis Interactome. Interestingly, functional modules tend to be formed by proteins involved in the same stage of symbiosis [see Additional file 2] suggesting that distinct symbiosis-stages are organized and coordinated as distinct functional modules. Therefore, the incorporation of modules apart from providing another structural dimension to the Symbiosis Interactome also allows the prediction of both protein function and the symbiosis-stage a novel component may participate (Fig. 3c). This highlight both the quality of the network and the functional modules we predicted as guide for direct experimental validation. The final 'Symbiosis Interactome network', therefore, hosts the organization of the Symbiosis Interactome into functional interactions and modules, and constitutes the first attempt toward the representation of this complex biological process (Fig. 4).

Novel predicted components include many conserved proteins of unknown functions and others participating in a variety of cellular processes (Fig. 4). Novel proteins may represent false negatives components not identified by current experimental techniques perhaps because they are highly specialized components or maybe recruited to the Symbiosis Interactome under specific conditions that have escaped from detection and are therefore absent from our 'classical-known' preliminary data. Our experimental results yielded a preliminary notable success (3 positive cases out of 4 proteins tested experimentally) for predicting novel *S. meliloti*-*M. sativa *symbiotic components by using our computational approach. The results also provide tempting clues in regard to the predictive potential of our approach for hypothesis generation and guiding future experimental validation. For example, the two module-network scenarios presented here suggest high accuracy at predicting novel components and functional modules. Furthermore, high scored interactions based on our probability scores are experimentally validated as opposed to low quality interactions for which we could not find any direct experimental evidence, at least not for the gene *Q92TC2 *tested here. For this particular protein and the remaining 259 non-tested novel proteins, it is difficult to determine how many of them could be really involved in this complex biological process. It has been described that mutations in some bacterial nodulation genes do not have any influence in the symbiotic properties of the bacteria. For example, *S. meliloti *cells mutated in *fixT *gene are not affected in nodulation with *M. sativa *host plants [51]. The expression of this fixation gene is regulated by FixH protein, which is essential for nodulation (mutations in *fixH *gives a Fix^- ^phenotype, that is, non-fixing nodules). It has been suggested that some nodulation proteins could have a role in symbiosis when the expression of essential proteins is blocked. In the same manner, there are proteins that could be essential for nodulation in special situations, such as biotic and abiotic stress. In addition, there are proteins that could be involved in the symbiotic competitiveness of the rhizobial strain. Finally, another alternative explanation is that the potential involvement of the gene *Q92TC2 *in symbiosis might be compensated by other genes performing similar functions. Indeed, a gene family analysis by using sequence similarity clustering through the MCL algorithm [35] (see methods) revealed an intriguing gene family expansion in this particular case (31 genes in this family), whereas in the other 3 mutated genes we do not observe such drastic family expansions (with 1 (singleton family), 3, and 14 gene family members, for the genes *Q92P53*, *etfB1*, and *msbA1*, respectively). This interesting result suggests that other members of this large gene family might rescue its potential role in symbiosis through the establishment of backup circuits, such as occurs in other well studied model organisms [52]. There is evidence of direct backup compensation between gene duplicates with overlapping functions where one gene can cover for the loss of its paralogue, and sometimes these compensations occur only for certain functions under given conditions [52]. In all these situations, the single mutation of these genes in conventional laboratory conditions would not be the best experiment to assess their role in symbiosis. We believe this novel finding supports the model of network robustness through gene duplication [53], and it also has very interesting implications regarding the selection of the right candidate genes and experimental method in future validation studies.

While the functional network presented here provides valuable clues about the components of the bacterial Symbiosis Interactome, the main limitation of our study is the lack of experimental information on PPIs which made us to consider as input only computationally derived functional genomics data. Integration of computational approaches with recently published [54] and future experimental interaction data would likely improve the quality of our network and the prediction of novel components. This can be done by using Bayesian or probabilistic models shown to result in accurate confidence scoring systems [[26,27,55], Peregrín-Alvarez JM, Xiong X, Su C, Parkinson J: The modular organization of protein interactions in *Escherichia coli*, submitted]. Furthermore, although we believe we have been very flexible by allowing interactions between proteins with potential phenotypic profiles and not directly interacting with the giant-central network component, our Symbiosis Interactome network can still serve as a platform to add other interactions and components potentially involved in symbiosis. For example, we can choose other proteins with other interesting phenotypic profiles to extend our network such as those profiles showing homologs in other symbionts and/or pathogenic species since these bacteria often use the same core molecular mechanism to maintain their associations with hosts [56]. Future analyses will also include further network extensions based on recently characterized symbiosis components [57-59], inclusion of other interesting phenotypic profiles (see above), a larger-scale experimental validation of the novel components predicted to be involved in symbiosis, and further analyses of the components and pathways involved in host-microbe, and host (i.e. plant) interactions. Finally, through an iterative process, novel Symbiosis Interactome components once experimentally confirmed, can be then added to the known set, potentially increasing the list of novel components and finally revealing the complete picture of the Symbiosis Interactome network.

## Conclusion

The essential contribution of symbiosis to understand host-microbe interactions underscores the importance of further studying the structure and organization of the Symbiosis Interactome. Here we presented a novel 'systems-based model' that provided for the very first time new insights into the functional organization of the *S. meliloti *Symbiosis Interactome and the necessary framework on which to build, in an iterative manner, to further our understanding of symbiosis. We have identified 263 potential novel symbiosis components, and have demonstrated experimentally the participation of novel proteins involved in this important process. These novel proteins might not be essential for symbiosis but still determinant for the microbe-plant interaction since most of the essential components for this process have been described through decades of effort. Understanding the biology of this important model organism is essential not only for having a network view of how this biological process functions at a molecular level but also for the development of anti-microbial drugs since many of the proteins and modules involved in bacterial-symbiosis may be conserved, and thus, performing similar functions, in other microbial pathogens [56]. Furthermore, we can use our network as a template to derive other Symbiosis Interactome networks for other bacteria-related species which is particularly important given the difficulty and cost of obtaining high throughput screens. Those maps should provide an useful starting point for predicting functional interactions and modules, and the function of unknown proteins. It remain to be seen which of these interactions and components do indeed occur and what is the specific role they play in each of these organisms. We believe that this model adds a new view and dimension to our understanding of host-microbe interactions, and can be extended to study other complex biological processes such as those involved in diseases.

## Methods

### Literature curation

An initial list of proteins known to be involved in the *Rhizobium*-Legume symbiosis was obtained and manually curated using PubMed, Google, journal-specific searches, and literature reviews and citations. We have called this list the 'classical-known' set.

### Network generation

We used *S. meliloti *genome context data from the PROLINKS [13] and STRING [14] databases. While both databases use the same genome context methods to derive functional linkages they both differ in the statistical procedures and scoring systems they use to provide high quality interactions. We reasoned that the overlap between both databases (intersection) represents interactions more likely to be true positives, and that the union of both databases represents a dataset with higher coverage (see below). We used all medium-to-high confidence functional linkages provided by the STRING database. From PROLINKS database we used those functional linkages in *S. meliloti *over 0.6 confidence. This cut-off provided a true positive rate similar to the one obtained by using the medium-to-high confidence data from the STRING database. The genome context data obtained from these two databases were combined into two single non-redundant datasets: one based on the overlapping between these two databases (the intersection dataset), and another one based on the union of the databases (the union dataset).

The confidence scores associated to each functional linkage provided by the original STRING and PROLINKS databases were re-scored according to the following criteria: STRING provides unified scores representing the confidence of a given functional linkage. The bigger the score, the more reliable the interaction. We reasoned that those interactions present in both databases are the most reliable ones, and we tested it by calculating ROC curves (see below). STRING scores were transformed into a scoring scale 0 – 0.5, the closer to 0.5, the bigger the confidence of the interaction. PROLINKS provides independent confidence scores for each applied independent genome context method. The scores were combined into an unified score by summing all confidence scores for a particular functional linkage and transforming the resulting number to a 0 – 0.5 scale. This procedure resulted in a 0 – 1 confidence score for those functional linkages present in both databases (the intersection data set) and a 0 – 0.5 confidence score for those interactions present in only one of the databases.

### ROC analyses

The validity of our re-scoring approach and the integrated networks was tested by calculating Receiving Operating Curves (ROC) and the Area Under the Curve (AUC) of the intersection, union, PROLINKS and STRING data sets as a measure of accuracy.

To be able to calculate accurate ROC curves and AUCs it is crucial to complement a positive gold standard set with a negative one. Because a reference set of known interactions is not available for *S. meliloti*, here we consider as positive set those functional linkages belonging to the same COGs functional category [37,60]. The construction of a negative set is rather problematic because it is impossible to be sure that two proteins do not interact. However, by using those pairs of proteins that are present in different COGs functional categories and do not colocalize in the same cell compartment it is possible to make a list of protein pairs that are unlikely to interact, thus representing a good approximation to a negative set. COGs annotations were mapped to functional linkages and the periplasmic location of all the proteins was predicted (see below).

### Subcellular localization

The periplasmic location of all *S. meliloti *proteins was predicted using the SIGCLEAVAGE software [38]. The proteins were considered as periplasmic if they contained at least one predicted signal sequence within 50 residues from the N terminus. The proteins that did not contain any signal sequence throughout the entire sequence were considered cytoplasmic, and the remaining proteins were not classified.

### Gene mapping

The protein IDs of the functional linkage data from the intersection and union networks were converted to gene names using UNIPROT database [61] and these were used to map the list of classical-known proteins in *S. meliloti*.

### Phenotypic profiling

For each *S. meliloti *sequence, a BLASTP [62] search was performed against 200 complete genome datasets. Both *S. meliloti *and other complete genomes were downloaded from the COGENT database [63] [see Additional file 2]. Homologs for each *S. meliloti *protein were determined based on a raw bit score threshold of 50, and were used to generate phenotypic profiles as follow: the complete genomes were manually curated and assigned to the following 8 phenotype categories [see Additional file 2] using PubMed, Google, and other web-specific searches: C, root colonizing bacteria; Fn, nitrogen-fixing bacteria in symbiosis with plants; Fl, free living nitrogen-fixing bacteria; P, pathogen; Pp, plant pathogen; S, soil cohabitant; Sy, symbiont/commensal; and O, other organisms. The only restriction for categorizing was that the genome in question have to be classified into one category only, the one with the most relevant phenotype for the study of symbiosis [see Additional file 2]. For example, if a genome could be classified as C and S, we considered only the category C because all C are also category S; or if a genome could be classified as P and S, P was considered more important for our analysis and thus classified as P only.

We then built phylogenetic profiles [18] for each *S. meliloti *protein and mapped the phenotypic data on top of the phylogenetic profiles yielding what we term 'phenotypic profiles'. For example, a protein with a phenotypic profile "FnFl" stands for a protein with homologs in plant nitrogen fixing bacteria and free-living nitrogen fixing bacteria only, thus representing a protein that may be potentially involved in symbiosis.

### Network analyses

Unless otherwise noted network analyses were performed using Perl scripts developed in house. The degree (*k*) of a node (protein) in an interaction network is defined by the number of interactions of the node with other nodes in the network. For a node of degree k, its clustering coefficient (CC) is defined as 2N/k(k-1), where N is the number of interactions between the node's k neighbors and k(k-1)/2 is the number of possible interactions between its neighbors. A CC of 1 means that all the neighbors of a node are fully interconnected. The shortest path length between two nodes in the network is the number of edges in a shortest path connecting them. The shortest path length is infinity if there are no paths between two nodes. Network diameters were obtained using Pajek [64], and cluster coefficients and shortest path lengths were obtained using tYNA [65].

To act as controls, random networks were created by randomly selecting equal numbers of proteins (compared with the comparator network) from the *S. meliloti *network and randomly connecting them with equal numbers of interactions.

For network comparisons with *E. coli *interaction data sets, experimental PPIs from various large- and small-scale experiments in *E. coli *were collected from the Database of Interacting Proteins (DIP) [31]. PPIs from DIP were divided into two main categories small-scale experiments and large-scale TAP assays. A third large-scale PPI data set was obtained from a recent large-scale pull down study [7]. The Interologs approach [22] was then performed by applying BLAST [62] to the *S. meliloti *proteome as query versus the *E. coli *proteome as database. Then we calculated *S. meliloti *orthologs (defined by BLAST best reciprocal hits with a cut-off e-value of 10^-10^) and mapped the *E. coli *interactomes mentioned above to derive *S. meliloti *interologs. We considered a functional interaction to be preserved in a genome if both interacting proteins have detectable orthologs. When comparing different networks a network similarity (NS) measure was devised:



where A and B represents different networks, S_AB _the similarity (i.e. the frequency of common interactions) of A versus B, and S_BA _the similarity of B versus A.

Networks were visualized using Biolayout [66] and Cytoscape [67].

### Detection of functional modules

We identified highly connected functional modules operating within the intersection *S. meliloti *network by using the Markov Cluster (MCL) algorithm [35]. MCL was applied to our *S. meliloti *network by testing several inflation operators, and settling on values that provided the highest clusters size, and the best overlap (semantic similarity) [68] of the computed clusters with the functional categories of the highly curated database COGs [37].

To compute the significant of finding specific COGs modules, a p-value for each module was calculated based on the distribution of 10,000 random module sets of the same size (assuming a normal distribution) and our module predictions, therefore, representing the probability of seeing such modules at chance. COGs categories with general function prediction, unknown or unassigned were not considered in this analysis. Only modules with at least three components with COGs assignments were statistically computed.

### Prediction of functional annotation and stage of symbiosis

Predictions of functional annotation and the stage of symbiosis were performed using enrichment of COGs terms in functional modules (see above). Module prediction for a protein employed the predicted functional modules and derived COGs/symbiosis-stage annotations for the target proteins based on the highest percentage of common COGs/symbiosis-stage terms among the different components of the functional module. Correct COGs/symbiosis-stage assignments additionally required at least 20% of the interaction module components to have the same COGs/symbiosis-stage category. Two measures of stringency were employed: high stringency predictions required the majority of interaction module components to be assigned to the same COGs/symbiosis-stage category; low stringency predictions only required any of the interaction module components to possess the same COGs/symbiosis-stage category (albeit with the additional proviso that at least 20% of the module partners were so annotated). To measure the accuracy of module predictions we used a leave-one-out (LOO) cross-validation procedure, i.e. only proteins which itself and one of its module components possessed an annotation were used in cross-validation. The LOO method randomly selects a protein and compares its known annotation with that predicted by the functional module method.

### Gene family analyses

Gene family predictions for the *S. meliloti *dataset were obtained from COGENT database [63] through the use of the MCL algorithm [35].

#### *S. meliloti *mutants

*S. meliloti *mutants were obtained from a Mini-Tn*5 *transposon library constructed in the Lehrstuhl für Genetik (Bielefeld University, Germany) [47]. Based on four network scenarios (see results) we selected the following *S. meliloti *mutants for experimental validation of our approach: 2011mTn*5*STM.3.02.D12_transposon(etfB1), 2011mTn*5*STM.4.10.F09_transposon(Q92P53), 2011mTn*5*STM.3.08.C10_transposon(Q92TC2), and 2011mTn*5*STM.1.06.E11_transposon (msbA1).

#### Nodulation assays

Seeds of alfalfa (*Medicago sativa *L. ecotype. Aragon) were surface sterilised on 70% ethanol for 10 minutes, exhaustively washed on distilled water and placed in water-agar plates for 36 hours at 22°C in the dark. 0.5–1 cm root pre-germinated seedlings were carefully transferred to squared plates containing a slope of BNM-agar medium [69]. Seedlings were inoculated with 100 μl of an overnight culture of *S. meliloti *mutants or the strain 1021 as control. The lower part of the plate was covered with black paper in order to avoid the roots getting exposed to light. Plates were placed on an Ibercex G-28 plant growth cabinet at 22°C with 16 hours photoperiod. Plants were taken out of the plates at 28 days post-inoculation (dpi) for nodule analyses (counting, size, color, etc). Three independent experiments with 50 plants per experiment were done (150 plants in total). General aspects of plants were also analysed.

## Authors' contributions

Literature searches, curation and the generation of the classical-know list were performed by R-L.I and C.MA. Phenotypic profiles were devised and curated by P-A.JM, R-L.I, C.MA, P.AJ. and C.FM. Experiments were devised and performed by R-L.I, C.MA. and D.M. The generation and curation of the networks described here were jointly performed by P-A.JM, R-L.I and C.MA. Network and computational analyses were performed by P-A.JM. The manuscript was jointly drafted by P-A.JM, R-L.I, C.MA, P.AJ. and C.FM. The study was conceived and supervised by P-A.JM. All authors approved the final version of the manuscript.

## Supplementary Material

Additional file 1 **Network accuracy, scale-free topology, and conservation versus connectivity analysis**. (A) To assess the performance of our re-scoring method we calculated ROC curves and AUCs for the *S. meliloti *intersection, union, PROLINKS [13] and STRING [14] networks. (B) The scale-free topology of the *S. meliloti *and Symbiosis Interactome networks. The connectivity distribution (k) per protein is plotted as a function of frequency, P(k). R, Pearson's correlation coefficient. (C) Relationship between protein conservation and connectivity within the *S. meliloti *and Symbiosis Interactome network. High conservation is defined as those proteins with homologs in more than 150 genomes (out of 200), and low conservation for proteins with homologs in less than 25 genomes. High connectivity proteins are defined as those with more than 10 connections and low connectivity for those ones with less than 3 connections. Click here for file

Additional file 2 **Supplementary Tables**. **Table 1**. List of genes known to be involved in the *Rhizobium*-Legume interaction (the 'classical-known' set); **Table 2**. List of the 200 complete genomes used in this study and the phenotype categories assigned to them. Complete genomes where obtained from the COGENT database [63]. Group and chosen group stands for the preliminary and final phenotypic categories, respectively, that were further used to build the phenotypic profiles; **Table 3**. The distribution of phenotypic profiles for the list of classical-known *S. meliloti *proteins; **Table 4**. Prediction of functional modules in the *S. meliloti *network. Functional modules were predicted using the MCL algorithm [35]. Size represents the number of components within the module. Module annotation was obtained by measuring the overlap of COGs categories [37] (in percentage) among the module components, otherwise module annotation was assigned to blank. COG category codes are provided in Fig. 4. P-values were calculated based on expectation using 10,000 random modules of equal size. * = p-value < 0.01; ** = p-value < 0.001. Modules with no (blank) p-value assignment (due to the lack or poor COGs annotation statistics were not computed) were considered as potential novel functional modules for the purpose of this study;**Table 5**. List of classical-known and novel proteins predicted to be involved in *S. meliloti*-Legume interaction in this study. The proteins present in our Symbiosis Interactome Interactome are represented. Gene and ORF names, and annotations were obtained from UNIPROT [61]. Colour code for classical-known proteins indicate the stage of symbiosis proteins are involved in, as in Fig. 4. Proteins with regulatory functions are represented by gene names with bold letters. Profile represents phenotypic profiles. Module stands for the functional modules predicted by MCL clustering. FC represents COGs functional categories as in Fig. 4. Location represents the predicted subcelullar localization. SC and GC represent the connectivity (i.e. number of interactors) of the protein in the Symbiosis Interactome and *S. meliloti *network, respectively. Click here for file

Additional file 3 **List of 440 classical-known and novel proteins, and 1,041 functional interactions predicted to be part of the Symbiosis Interactome network**. Proteins are plotted as gene names according to UNIPROT. Scores represent probability of interactions. Click here for file
