# Supplementary figures and images for: The Symbiosis Interactome: a computational approach reveals novel components, functional interactions and modules in Sinorhizobium meliloti
Source: BMC Syst Biol. 2009 Jun 16;3:63. doi: 10.1186/1752-0509-3-63 (PMC2701930; doi:10.1186/1752-0509-3-63)

A

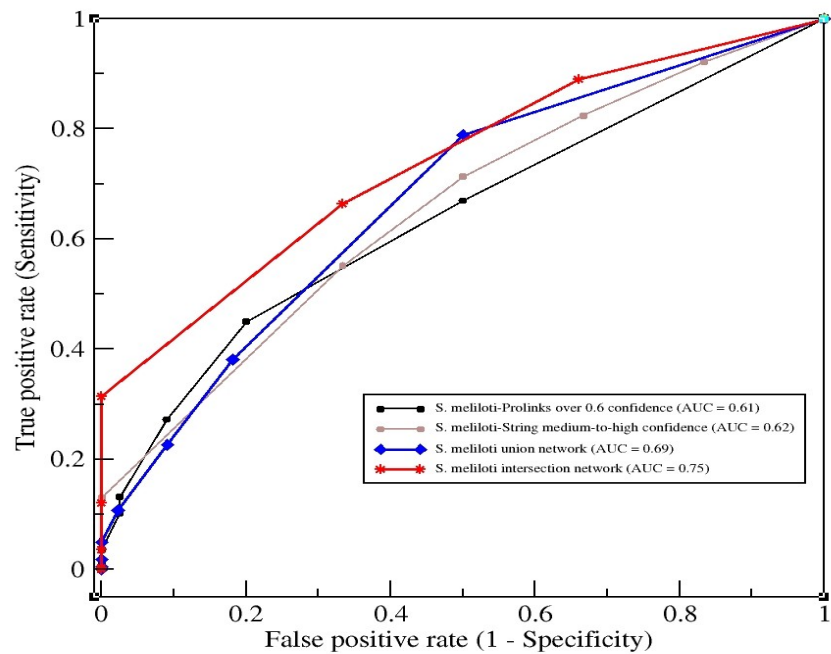

B

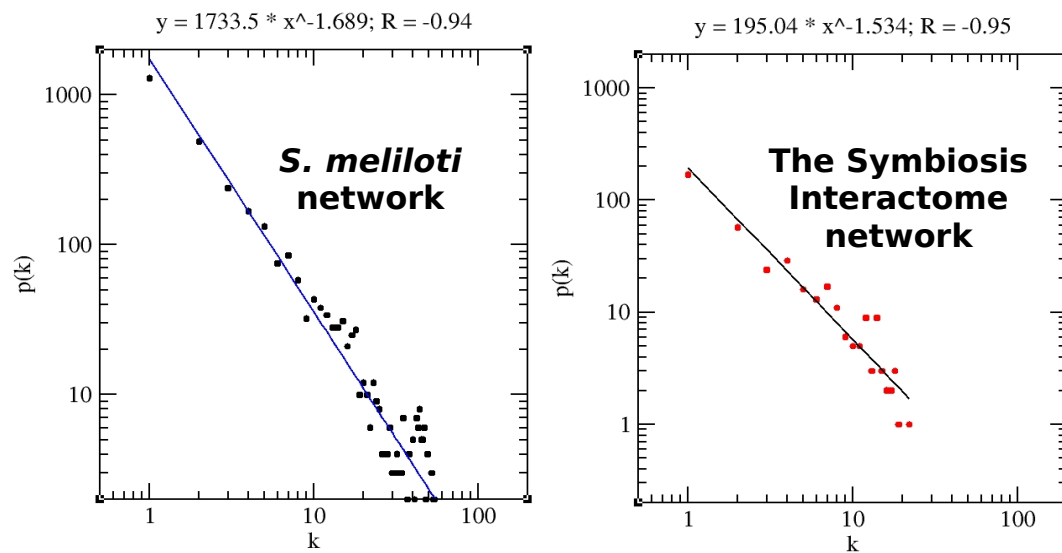

C

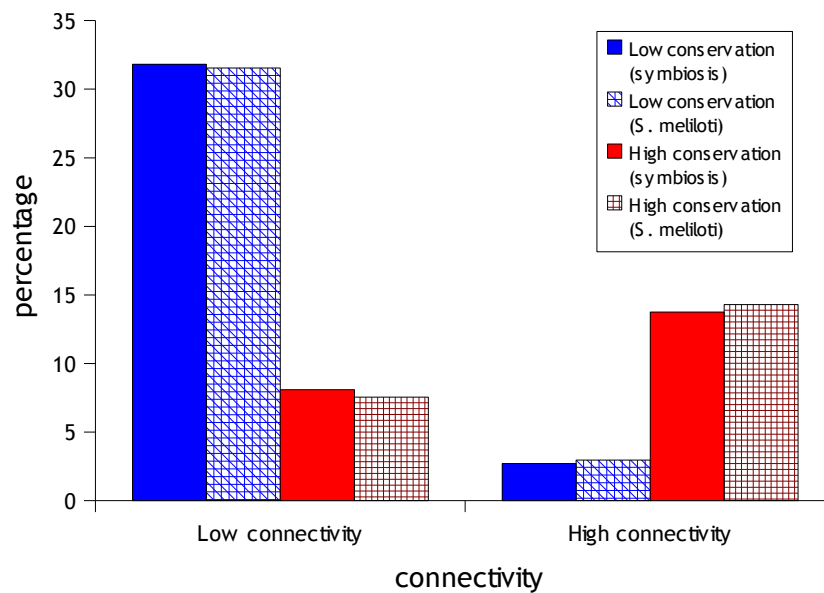

Supplement: Additional file 1 — Network accuracy, scale-free topology, and conservation versus connectivity analysis. (A) To assess the performance of our re-scoring method we calculated ROC curves and AUCs for the S. meliloti intersection, union, PROLINKS [13] and STRING [14] networks. (B) The scale-free topology of the S. meliloti and Symbiosis Interactome networks. The connectivity distribution (k) per protein is plotted as a function of frequency, P(k). R, Pearson's correlation coefficient. (C) Relationship between protein conservation and connectivity within the S. meliloti and Symbiosis Interactome network. High conservation is defined as those proteins with homologs in more than 150 genomes (out of 200), and low conservation for proteins with homologs in less than 25 genomes. High connectivity proteins are defined as those with more than 10 connections and low connectivity for those ones with less than 3 connections. [file 1752-0509-3-63-S1.pdf]
