# Supplementary material for: The Symbiosis Interactome: a computational approach reveals novel components, functional interactions and modules in Sinorhizobium meliloti
Source: BMC Syst Biol. 2009 Jun 16;3:63. doi: 10.1186/1752-0509-3-63 (PMC2701930; doi:10.1186/1752-0509-3-63)
Supplement: Additional file 2 — Supplementary Tables. Table 1. List of genes known to be involved in the Rhizobium-Legume interaction (the 'classical-known' set); Table 2. List of the 200 complete genomes used in this study and the phenotype categories assigned to them. Complete genomes where obtained from the COGENT database [63]. Group and chosen group stands for the preliminary and final phenotypic categories, respectively, that were further used to build the phenotypic profiles; Table 3. The distribution of phenotypic profiles for the list of classical-known S. meliloti proteins; Table 4. Prediction of functional modules in the S. meliloti network. Functional modules were predicted using the MCL algorithm [35]. Size represents the number of components within the module. Module annotation was obtained by measuring the overlap of COGs categories [37] (in percentage) among the module components, otherwise module annotation was assigned to blank. COG category codes are provided in Fig. 4. P-values were calculated based on expectation using 10,000 random modules of equal size. * = p-value < 0.01; ** = p-value < 0.001. Modules with no (blank) p-value assignment (due to the lack or poor COGs annotation statistics were not computed) were considered as potential novel functional modules for the purpose of this study;Table 5. List of classical-known and novel proteins predicted to be involved in S. meliloti-Legume interaction in this study. The proteins present in our Symbiosis Interactome Interactome are represented. Gene and ORF names, and annotations were obtained from UNIPROT [61]. Colour code for classical-known proteins indicate the stage of symbiosis proteins are involved in, as in Fig. 4. Proteins with regulatory functions are represented by gene names with bold letters. Profile represents phenotypic profiles. Module stands for the functional modules predicted by MCL clustering. FC represents COGs functional categories as in Fig. 4. Location represents the predicted subcelullar localization. SC [file 1752-0509-3-63-S2.pdf]

Table 1. List of genes known to be involved in *Rhizobium*-Legume symbiosis.

Annotation	Stage of symbiosis	Gene
General	Nitrogen fixation	Fix, nif
General	Nodulation	Nod, noe, nol
General	Exopolysaccharide synthesis	exo, exs, exp
General	Glucane synthesis	NdvA, nov, cgm
General	Lipopolysaccharide synthesis	lps
General	Capsular polysaccharide synthesis	cps
General	KPS synthesis	rkp
General	Regulator of nod and exopolysaccharide genes	syrM
General	Motility and competitivity	VisN, visR
General	Gene regulator	mucR
General	Regulator	NifA-rpoN
General	Bacteroid sensor	bacA
Metabolism	Hydrogenase synthesis	hup
Metabolism	Nitrogen metabolism regulation	ntr
Metabolism	Nitrogen metabolism in *Bradyrhizobium japonicum*	nap, nir, nor, nos
Metabolism	Fatty acid synthesis	acp
Suspicious	Nodule formation efficiency	nfe
Suspicious	Dicarboxilic acid transport regulator	dct
Suspicious	Rhizopine synthesis	mos
Suspicious	Rhizopine catabolism	moc
Suspicious	Porine synthesis	rop

Table 2. Complete genomes and phenotype categories

Species	Cogent Code	Group	Chosen group
Aquifex aeolicus	AAEO-VF5-01	O	O
Acinetobacter calcoaceticus	ACIN-DP1-01	S/P	P
Archaeoglobus_fulgidus	AFUL-DSM-01	O	O
Anopheles_gambiae	AGAM-PES-01	O	O
Ashbya_gossypii	AGOS-XXX-01	O	O
Aeropyrum_pernix	APER-XK1-01	O	O
Arabidopsis_thaliana	ATHA-XXX-01	O	O
Agrobacterium tumefaciens	ATUM-C58-01	Pp	Pp
Bacillus anthracis	BANT-AME-01	C/S/P	C
Buchnera aphidicola	BAPH-XBP-01	Sy	Sy
Buchnera aphidicola	BAPH-XSG-01	Sy	Sy
Bdellovibrio bacteriovorus	BBAC-100-01	Sy	Sy
Bordetella bronchiseptica	BBRO-252-01	P	P
Borrelia burgdorferi	BBUR-B31-01	P	P
Bacillus cereus	BCER-579-01	C/S/P	C
Bacillus cereus	BCER-987-01	C/S/P	C
Blochmannia floridanus	BFLO-XXX-01	Sy	Sy
Bacteroides fragilis	BFRA-H46-01	P	P
Bacillus halodurans	BHAL-C12-01	C/S	C
Bartonella henselae	BHEN-HOU-01	P	P
Bradyrhizobium japonicum	BJAP-USD-01	Fn	Fn
Bacillus licheniformis	BLIC-580-01	C/S	C
Bifidobacterium longum	BLON-NCC-01	Sy	Sy
Burkholderia mallei	BMAL-344-01	P	P
Brucella melitensis	BMEL-M16-01	P	P
Bordetella parapertussis	BPAR-253-01	P	P
Bordetella pertussis	BPER-251-01	P	P
Burkholderia pseudomallei	BPSE-243-01	C/S/P	C
Bartonella quintana	BQUI-TOU-01	P	P
Bacillus subtilis	BSUB-168-01	Pp/S	Pp
Brucella suis	BSUI-133-01	P	P
Bacteroides thetaiotaomicron	BTHE-VPI-01	Sy/P	Sy
Buchnera sp.	BUCH-APS-01	Sy	Sy
Clostridium acetobutylicum	CACE-ATC-01	S	S
Caenorhabditis_briggsae	CBRI-XXX-01	O	O
Coxiella burnetii	CBUR-RSA-01	S/P	P
Chlamydophila caviae	CCAV-GPI-01	P	P
Caulobacter crescentus	CCRE-XXX-01	O	O

Corynebacterium diphtheriae	CDIP-129-01	P	P
Corynebacterium efficiens	CEFF-YS3-01	C/S	C
Caenorhabditis_elegans	CELE-XXX-01	O	O
Candida_glabrata	CGLA-138-01	O	O
Corynebacterium glutamicum	CGLU-XXX-01	C/S	C
Campylobacter jejuni	CJEJ-NCT-01	S/P	P
Cyanidioschyzon_merolae	CMER-10D-01	O	O
Cryptosporidium_parvum	CPAR-TII-01	O	O
Clostridium perfringens	CPER-X13-01	S/P	P
Chlamydia pneumoniae	CPNE-AR3-01	P	P
Chlamydia pneumoniae	CPNE-CWL-01	P	P
Chlamydia pneumoniae	CPNE-J13-01	P	P
Chlorobium tepidum	CTEP-TLS-01	O	O
Clostridium tetani	CTET-E88-01	S/P	P
Chlamydia trachomatis	CTRA-MOP-01	P	P
Chlamydia trachomatis	CTRA-SVD-01	P	P
Chromobacterium violaceum	CVIO-472-01	S/P	P
Debaryomyces_hansenii	DHAN-767-01	O	O
Drosophila_melanogaster	DMEL-XXX-02	O	O
Desulfotalea psychrophila	DPSY-V54-01	S	S
Deinococcus radiodurans	DRAD-XR1-01	S	S
Desulfovibrio vulgaris	DVUL-HIL-01	S	S
Erwinia carotovora subsp. atroseptica	ECAR-043-01	Pp/S	Pp
Escherichia coli	ECOL-CFT-01	P	P
Escherichia coli O157:H7	ECOL-EDL-01	P	P
Escherichia coli	ECOL-MG1-01	Sy	Sy
Escherichia coli 0157:H7	ECOL-RIM-01	P	P
Encephalitozoon_cuniculi	ECUN-XXX-01	O	O
Enterococcus faecalis	EFAE-V58-01	C/Sy/P	Sy
Fusobacterium nucleatum	FNUC-ATC-01	P	P
Geobacter sulfurreducens	GSUL-PCA-01	S	S
Gloeobacter violaceus	GVIO-421-01	S	S
Halobacterium_sp.	HALO-NRC-01	O	O
Helicobacter hepaticus	HHEP-449-01	P	P
Haemophilus influenzae	HINF-KW2-01	P	P
Helicobacter pylori	HPYL-266-01	P	P
Helicobacter pylori	HPYL-J99-01	P	P
Homo_sapiens	HSAP-XXX-03	O	O
Kluyveromyces_lactis	KLAC-210-01	O	O
Listeria innocua	LINN-CLI-01	S	S
Leptospira interrogans	LINT-130-01	P	P

Leptospira interrogans	LINT-566-01	P	P
Lactobacillus johnsonii	LJOH-533-01	Sy	Sy
Lactococcus lactis	LLAC-IL1-01	Sy	Sy
Listeria monocytogenes	LMON-365-01	S/P	P
Listeria monocytogenes	LMON-854-01	S/P	P
Listeria monocytogenes	LMON-858-01	S/P	P
Listeria monocytogenes	LMON-EGD-01	S/P	P
Lactobacillus plantarum	LPLA-WCF-01	Sy	Sy
Legionella pneumophila	LPNE-LEN-01	S/P	P
Legionella pneumophila	LPNE-PHI-01	S/P	P
Leifsonia xyli subsp. xyli	LXYL-B07-01	Pp	Pp
Methanosarcina_acetivorans	MACE-C2A-01	O	O
Mycobacterium bovis	MBOV-AF2-01	P	P
Methylococcus capsulatus	MCAP-BAT-01	S	S
Mycoplasma gallisepticum	MGAL-RLO-01	P	P
Mycoplasma genitalium	MGEN-G37-01	P	P
Mycoplasma hyopneumoniae	MHYO-232-01	P	P
Methanococcus_jannaschii	MJAN-DSM-01	O	O
Methanopyrus_kandleri	MKAN-AV1-01	O	O
Mycobacterium leprae	MLEP-XTN-01	P	P
Mesorhizobium loti	MLOT-MAF-01	Fn	Fn
Methanococcus maripaludis	MMAR-XS2-01	Fl	Fl
Methanosarcina_mazei	MMAZ-GO1-01	O	O
Mycoplasma mobile	MMOB-63K-01	P	P
Mus_musculus	MMUS-XXX-02	O	O
Mycoplasma mycoides	MMYC-G1T-01	P	P
Mycoplasma penetrans	MPEN-HF2-01	P	P
Mycoplasma pneumoniae	MPNE-M12-01	P	P
Mycoplasma pulmonis	MPUL-UAB-01	P	P
Mannheimia succiniciproducens	MSUC-55E-01	Sy	Sy
Methanobacterium_thermoautotrophicum	MTHE-DEL-01	O	O
Mycobacterium tuberculosis	MTUB-CDC-01	P	P
Mycobacterium tuberculosis	MTUB-H37-01	P	P
Neurospora_crassa	NCRA-XX3-01	O	O
Nanoarchaeum equitans	NEQU-N4M-01	P	P
Nitrosomonas europaea	NEUR-718-01	S	S
Nocardia farcinica	NFAR-152-01	S/P	P
Neisseria meningitidis	NMEN-MC5-01	P	P
Neisseria meningitidis	NMEN-Z24-01	P	P
Nostoc (Anabaena) sp.	NOST-PCC-01	Fl/S	Fl
Oceanobacillus iheyensis	OIHE-HET-01	O	O

Pyrococcus_abyssi	PABY-GE5-01	O	O
Propionibacterium acnes	PACN-202-01	Sy	Sy
Pyrobaculum_aerophilum	PAER-IM2-01	O	O
Pseudomonas aeruginosa	PAER-PAO-01	Pp/S/P	Pp
Phytoplasma asteris	PAST-XOY-01	Pp	Pp
Parachlamydia sp.	PCHL-E25-01	P	P
Plasmodium_falciparum	PFAL-3D7-01	O	O
Pyrococcus_furiosus	PFUR-638-01	O	O
Porphyromonas gingivalis	PGIN-W83-01	P	P
Pyrococcus_horikoshii	PHOR-OT3-01	O	O
Pirellula sp.	PIRE-ST1-01	O	O
Photorhabdus luminescens	PLUM-TO1-01	Sy	Sy
Prochlorococcus marinus	PMAR-MED-01	O	O
Prochlorococcus marinus	PMAR-MIT-01	O	O
Prochlorococcus marinus	PMAR-SS1-01	O	O
Pasteurella multocida	PMUL-PM7-01	P	P
Pseudomonas putida	PPUT-KT2-01	C/S	C
Pseudomonas syringae pv. tomato	PSYR-DC3-01	Pp/S	Pp
Picrophilus_torridus	PTOR-790-01	O	O
Rickettsia conorii	RCON-MAL-01	P	P
Rhodopseudomonas palustris	RPAL-009-01	S	S
Rickettsia prowazekii	RPRO-MAD-01	P	P
Ralstonia solanacearum	RSOL-XXX-01	Pp/S	Pp
Rickettsia typhi	RTYP-144-01	P	P
Streptococcus agalactiae	SAGA-260-01	P	P
Streptococcus agalactiae	SAGA-NEM-01	P	P
Staphylococcus aureus	SAUR-252-01	P	P
Staphylococcus aureus	SAUR-476-01	P	P
Staphylococcus aureus VRSA	SAUR-MU5-01	P	P
Staphylococcus aureus MRSA	SAUR-MW2-01	P	P
Staphylococcus aureus MRSA	SAUR-N13-01	P	P
Streptomyces avermitilis	SAVE-XXX-01	C/S	C
Saccharomyces_cerevisiae	SCER-S28-01	O	O
Streptomyces coelicolor	SCOE-A32-01	C/S	C
Salmonella enterica serovar Typhi	SENT-CT1-02	P	P
Salmonella enterica serovar Typhimurium	SENT-LT2-01	P	P
Salmonella enterica	SENT-TY2-01	P	P
Shigella flexneri (serotype 2a)	SFLE-301-01	P	P
Shigella flexneri	SFLE-457-01	P	P
Sinorhizobium meliloti	SMEL-102-01	Fn	Fn
Streptococcus mutans	SMUT-UA1-01	P	P

Shewanella oneidensis	SONE-MR1-01	S	S
Streptococcus pneumoniae	SPNE-TIG-01	P	P
Streptococcus pneumoniae	SPNE-XR6-01	P	P
Schizosaccharomyces_pombe	SPOM-XXX-01	O	O
Streptococcus pyogenes	SPYO-394-01	P	P
Streptococcus pyogenes M18	SPYO-MGA-01	P	P
Streptococcus pyogenes M1	SPYO-SF3-01	P	P
Streptococcus pyogenes M3	SPYO-SSI-01	P	P
Streptococcus pyogenes M3	SPYO-XM3-01	P	P
Sulfolobus_solfataricus	SSOL-XP2-01	O	O
Sulfolobus_tokodaii	STOK-XX7-01	O	O
Synechococcus sp.	SYCC-WH8-01	O	O
Synechocystis sp.	SYNE-PCC-01	O	O
Symbiobacterium thermophilum	SYTH-863-01	Sy	Sy
Thermoplasma_acidophilum	TACI-DSM-01	O	O
Treponema denticola	TDEN-405-01	P	P
Thermosynechococcus elongatus	TELO-BP1-01	O	O
Thermotoga maritima	TMAR-MSB-01	O	O
Treponema pallidum	TPAL-NIC-01	P	P
Thermoanaerobacter tengcongensis	TTEN-MB4-01	O	O
Thermus thermophilus	TTHE-B27-01	O	O
Thermoplasma_volcanium	TVOL-GSS-01	O	O
Tropheryma whipplei	TWHI-TW0-01	S/P	P
Tropheryma whipplei	TWHI-TWI-01	S/P	P
Ureaplasma urealyticum	UURE-SV3-01	P	P
Vibrio cholerae	VCHO-N16-01	P	P
Vibrio parahaemolyticus	VPAR-RIM-01	P	P
Vibrio vulnificus	VVUL-YJ0-01	P	P
Wigglesworthia glossinidia brevipalpis	WGLO-BRE-01	P	P
Wolbachia pipientis	WPIP-WME-01	P	P
Wolinella succinogenes strain DSM 1740	WSUC-740-01	Sy	Sy
Xanthomonas axonopodis pv. citri	XAXO-306-02	Pp	Pp
Xanthomonas campestris pv. campestris	XCAM-AT3-01	Pp	Pp
Xylella fastidiosa	XFAS-9A5-01	Pp	Pp
Xylella fastidiosa	XFAS-XPD-01	Pp	Pp
Yarrowia_lipolytica	YLIP-B99-01	O	O
Yersinia pestis	YPES-CO9-01	P	P
Yersinia pestis	YPES-KIM-01	P	P
Yersinia pseudotuberculosis	YPSE-953-01	S/P	P

Table 3. Phenotypic profile distribution of classical-known proteins

Phenotypic profile	Frequency	%
CFlFnPpPSSyO	39	0.42
CFnPpPSSyO	16	0.17
***S. meliloti* specific**	5	0.05
FnPp	5	0.05
FlFnPpPSSyO	3	0.03
CFnPpPSSy	2	0.02
FnPpPSO	2	0.02
FlFnPpSSyO	2	0.02
CFnPpPSO	2	0.02
CFnPpSO	2	0.02
FnPpPSSyO	1	0.01
FnPpPSSy	1	0.01
FnFlPp	1	0.01
CFlFnPpPSSy	1	0.01
FnPSSyO	1	0.01
FnPS	1	0.01
FnPpS	1	0.01
Fn	1	0.01
CFnPpPS	1	0.01
FlFnPpPO	1	0.01
FnPpPO	1	0.01
FnPpP	1	0.01
CFlFnPpPSyO	1	0.01
FnFlPpPSSyO	1	0.01
Total	92	1

Table 4. Functional modules

Module#	Size	COG enrichment (%)	p-value
1	61	100 E	0**
2	43	100 G	0**
3	35	71 E	0**
4	34	88 G	0**
5	26	77 J	0**
6	26	100 E	0**
7	25		
8	24		
9	24		
10	22	100 E	0**
11	21	91 E	0**
12	21	100 G	0**
13	21	81 N	0**
14	19	66 C	0**
15	18	100 E	0**
16	18	88 N	0**
17	18	88 T	0**
18	16	73 M	0**
19	15	100 K	0**
20	14	100 G	0**
21	13	81 M	0**
22	13	100 L	0**
23	12	100 Q	0**
24	11		
25	11	100 E	0**
26	11	77 I	0**
27	11		
28	9	87 C	0**
29	9	100 C	0**
30	9	77 E	0**
31	9	85 C	0**
32	9	88 F	0**
33	9	80 I	0**
34	9		
35	8	33 K	0.13
36	8	100 C	0**
37	8	50 C	0.01
38	8	100 T	0**
39	8		
40	8	87 H	0**
41	8	75 F	0.0001**
42	8	33 E	0.13
43	8	87 O	0**
44	8	100 G	0**
45	8		
46	7	100 C	0**
47	7		
48	7	42 O	0.08
49	7	100 H	0**
50	7	100 L	0**
51	7	50 T	0.007*
52	7	42 O	0.08
53	7	40 H	0.08
54	7	14 U	1
55	7	66 S	0.0006**
56	7	100 K	0**
57	7	71 H	0.0006**
58	6	100 E	0**
59	6	100 P	0**
60	6	100 Q	0**
61	6	100 G	0**
62	6	100 E	0**
63	6		
64	6	80 G	0.0001**
65	6	83 P	0.0001**
66	6	83 G	0.0001**
67	6	66 C	0.0036*
68	6	100 J	0**
69	6	60 G	0.0036*
70	6	100 K	0**
71	5	80 P	0.0007**
72	5		
73	5	100 C	0**
74	5	40 O	0.34
75	5	100 E	0**
76	5	100 G	0**
77	5	100 E	0**
78	5		
79	5		
80	5		
81	5		
82	5		
83	5		
84	5		
85	5		
86	5		
87	5		
88	5		
89	5		
90	5		
91	5		
92	5		
93	5		
94	5		
95	5		
96	5		
97	5		
98	5		
99	5		
100	5	100 C	0**
101	5	75 O	0.0007**
102	5		
103	4		
104	4	100 P	0.0003**
105	4	75 C	0.01
106	4		
107	4		
108	4		
109	4		
110	4	100 H	0.0003**
111	4		
112	4	100 C	0.0003**
113	4		
114	4	75 G	0.01
115	4	100 T	0.0003**
116	4		
117	4		
118	4		
119	4		
120	4	75 C	0.01
121	4	100 T	0.0003**
122	4	100 K	0.0003**
123	4		
124	4	100 M	0.0003**
125	4		
126	4	75 Q	0.01
127	4	100 P	0.0003**
128	4	100 V	0.0003**
129	4	75 M	0.01
130	4	50 F	0.23
131	4	25 T	1
132	4		
133	4	66 O	0.01
134	4	75 H	0.01
135	4		
136	4	100 H	0.0003**
137	4	100 L	0.0003**
138	4	100 L	0.0003**
139	4		
140	4	100 E	0.0003**
141	4	75 G	0.01
142	4	75 O	0.01
143	4	75 E	0.01
144	4	50 L	0.23
145	4	100 G	0.0003**
146	4	50 E	0.23
147	4	25 M	1
148	4		
149	4		
150	4	25 S	1
151	4		
152	4	50 M	0.23
153	4		
154	4		
155	4	100 G	0.0003**
156	4	25 G	1
157	4	66 L	0.01
158	4	100 C	0.0003**
159	4	100 S	0.0003**
160	4	66 F	0.01
161	4	100 E	0.0003**
162	4	100 C	0.0003**
163	4	100 M	0.0003**
164	4	100 T	0.0003**
165	3		
166	3		
167	3	100 H	0.0028*
168	3	100 E	0.0028*
169	3	100 G	0.0028*
170	3	33 L	0.99
171	3	66 E	0.12
172	3		
173	3	100 C	0.0028*
174	3		
175	3		
176	3	100 H	0.0028*
177	3	66 E	0.12
178	3	100 H	0.0028*
179	3		
180	3		
181	3	33 J	0.99
182	3		
183	3		
184	3	66 F	0.12
185	3		
186	3		
187	3	100 P	0.0028*
188	3	100 L	0.0028*
189	3	100 L	0.0028*
190	3		
191	3		
192	3		
193	3		
194	3	66 U	0.12
195	3		
196	3	66 S	0.12
197	3		
198	3	33 J	0.99
199	3	100 C	0.0028*
200	3	66 E	0.12
201	3		
202	3	100 I	0.0028*
203	3	100 G	0.0028*
204	3		
205	3	66 I	0.12
206	3	66 L	0.12
207	3		
208	3		
209	3		
210	3	66 O	0.12
211	3	100 C	0.0028*
212	3	50 I	0.12
213	3	100 P	0.0028*
214	3		
215	3	100 L	0.0028*
216	3	100 P	0.0028*
217	3	66 L	0.12
218	3	33 L	0.99
219	3		
220	3	100 J	0.0028*
221	3	100 L	0.0028*
222	3		
223	3		
224	3	66 L	0.12
225	3		
226	3		
227	3		
228	3	100 T	0.0028*
229	3	33 V	0.99
230	3	33 I	0.99
231	3	33 O	0.99
232	3	33 S	0.99
233	3		
234	3	66 U	0.12
235	3		
236	3	100 G	0.0028*
237	3		
238	3		
239	3	66 T	0.12
240	3	66 I	0.12
241	3		
242	3	66 H	0.12
243	3	33 L	0.99
244	3	66 P	0.12
245	3		
246	3	66 G	0.12
247	3		
248	3		
249	3		
250	3	100 V	0.0028*
251	3	33 O	0.99
252	3	100 E	0.0028*
253	3	100 P	0.0028*
254	3		
255	3		
256	3	33 S	0.99
257	3	66 D	0.12
258	3		
259	3	100 Q	0.0028*
260	3		
261	3	33 V	0.99
262	3	33 S	0.99
263	3		
264	3	100 Q	0.0028*
265	3	100 L	0.0028*
266	3	100 M	0.0028*
267	3	33 U	0.99
268	3	100 Q	0.0028*
269	3	66 O	0.12
270	3	100 L	0.0028*
271	3	66 G	0.12
272	3	33 E	0.99
273	3	100 T	0.0028*
274	3		
275	3	100 O	0.0028*
276	3		
277	3	66 L	0.12
278	3	50 S	0.12
279	3	33 P	0.99
280	3	66 K	0.12
281	3		
282	3	66 J	0.12
283	3	100 E	0.0028*
284	3	33 T	0.99
285	3	100 V	0.0028*
286	3	33 K	0.99
287	3	50 M	0.12
288	3	66 M	0.12
289	3	33 G	0.99
290	3	33 M	0.99
291	3		
292	3	33 F	0.99
293	3	100 S	0.0028*
294	3	33 Q	0.99
295	3	33 L	0.99
296	3	33 S	0.99
297	3	66 G	0.12
298	3		
299	3	100 M	0.0028*
300	3	33 O	0.99
301	3	100 I	0.0028*
302	3	66 P	0.12
303	3	33 G	0.99
304	3		
305	3		
306	3		
307	3		
308	3		
309	3		
310	3		
311	3		
312	3		
313	3		
314	3		
315	3		
316	3		
317	3		
318	3		
319	3		
320	3		
321	3		
322	3		
323	3		
324	3		
325	3		
326	3		
327	3		
328	3		
329	3		
330	3	100 I	0.0028*
331	3		
332	3		
333	3	66 N	0.12
334	3		
335	3	33 S	0.99
336	3	100 E	0.0028*
337	3		
338	3	66 S	0.12
339	3	100 M	0.0028*
340	3	33 S	0.99
341	3		
342	3	33 L	0.99
343	3		
344	3		
345	3	100 K	0.0028*

Table 5. List of classical-known and novel proteins potentially involved in *S. meliloti* symbiosis.

ID/genename	Orf name	Profile	Module	FC	Location	Annotation	SC	GC
expA10	SMB21327	CFlFnPpPSSyO	M91	-	periplasmic	PUTATIVE DTDP-4-DEHYDRORHAMNOSE REDUCTASE (EC1.1.1.133)	4	6
expA9	SMB21326	CFlFnPpPSSyO	M91	-	periplasmic	PUTATIVE DTDP-D-GLUCOSE 4,6-DEHYDRATASE (EC4.2.1.46)	5	6
expA8	SMB21325	CFlFnPpPSSyO	M91	-	periplasmic	PUTATIVE DTDP-4-DEHYDRORHAMNOSE 3,5-EPIMERASE (EC5.1.3.13)	4	6
expA7	SMB21324	CFlFnPpPSSyO	M91	-	-	PUTATIVE GLUCOSE-1-PHOSPHATE THYMIDYLTRANSFERASE (EC2.7.7.24)	7	12
Q92YV1	SMA1391	CFnPpPSSyO	M73	-	-	Probable EtfB2 electron transport flavoprotein, beta subunit.	4	4
etfA1	SMC00728	CFnPpPSSyO	M73	C	periplasmic	PUTATIVE ELECTRON TRANSFER FLAVOPROTEIN ALPHA-SUBUNIT ALPHA-ETF FLAVOPROTEIN	6	7
etfB1	SMC00729	CFnPpPSSyO	M73	C	-	PUTATIVE ELECTRON TRANSFER FLAVOPROTEIN BETA-SUBUNIT BETA-ETF FLAVOPROTEIN SMALL SUBUNIT	5	5
etf	SMC02377	CFnPpPSSyO	M73	C	periplasmic	PROBABLE ELECTRON TRANSFER FLAVOPROTEIN-UBIQUINONE OXIDOREDUCTASE	4	4
Q92YV2	SMA1389	CFnPpPSSyO	M73	-	periplasmic	Probable EtfA2 electron-transport flavoprotein, alpha-subunit.	6	7
nifD	SMA0827	FlFnPpSSyO	M63	-	-	NifD nitrogenase Fe-Mo alpha chain (EC 1.18.6.1).	4	4
nifK	SMA0829	FlFnPpSSyO	M63	-	-	NifK nitrogenase Fe-Mo beta chain (EC 1.18.6.1).	5	5
nifH	SMA0825	FlFnPpPSSyO	M63	-	periplasmic	Nitrogenase iron protein (EC 1.18.6.1) (Nitrogenase component II) (Nitrogenase Fe protein) (Nitrogenase reductase).	3	3
nifE	SMA0830	FlFnPpSSyO	M63	-	-	Nitrogenase iron-molybdenum cofactor biosynthesis protein nifE.	4	4
nifX	SMA0831	FlFnPpSSyO	M63	-	-	NifX nitrogen fixation protein.	1	1
nifN	SMA0873	FlFnPpSSyO	M63	-	periplasmic	Nitrogenase iron-molybdenum cofactor biosynthesis protein nifN.	5	5
engA	SMB20995	CFlFnPpPSSyO	M5	-	-	GTP-binding protein engA.	7	14
rpsJ	SMC01310	CFlFnPpPSSyO	M5	J	cytoplasmic	PROBABLE 30S RIBOSOMAL PROTEIN S10	17	21
efp	SMC00357	CFlFnPpPSSyO	M5	J	periplasmic	PROBABLE ELONGATION FACTOR P PROTEIN	4	8
adk	SMC01288	CFlFnPpPSSyO	M5	F	periplasmic	PROBABLE ADENYLATE KINASE PROTEIN	10	10
htpG	SMB21183	CFlFnPpPSSyO	M5	-	periplasmic	Chaperone protein htpG (Heat shock protein htpG) (High temperature protein G).	1	2
fusA	SMC01312	CFlFnPpPSSyO	M5	J	-	PROBABLE ELONGATION FACTOR G PROTEIN	14	14
rho	SMC02796	CFnPpPSSyO	M5	K	periplasmic	PROBABLE TRANSCRIPTION TERMINATION FACTOR RHO PROTEIN	1	1
rpsI	SMC01803	CFlFnPpPSSyO	M5	J	periplasmic	PROBABLE 30S RIBOSOMAL PROTEIN S9	7	10
rpsD	SMC00485	CFlFnPpPSSyO	M5	J	-	PROBABLE 30S RIBOSOMAL SUBUNIT PROTEIN S4	14	23
rpsC	SMC01303	CFlFnPpPSSyO	M5	J	-	PROBABLE 30S RIBOSOMAL PROTEIN S3	18	18
rpsS	SMC01305	CFlFnPpPSSyO	M5	J	cytoplasmic	PROBABLE 30S RIBOSOMAL PROTEIN S19	16	16
rpsE	SMC01292	CFlFnPpPSSyO	M5	J	-	PROBABLE 30S RIBOSOMAL PROTEIN S5	18	18
secY	SMC01289	CFlFnPpPSSyO	M5	U	periplasmic	PROBABLE PREPROTEIN TRANSLOCASE TRANSMEMBRANE	18	29
rpsN	SMC01296	CFlFnPpPSSyO	M5	J	cytoplasmic	PROBABLE 30S RIBOSOMAL PROTEIN S14	7	7
rplL	SMC01318	CFlFnPpPSSyO	M5	J	periplasmic	PROBABLE 50S RIBOSOMAL PROTEIN L7/L12 (L8)	1	1
rpsL	SMC01314	CFlFnPpPSSyO	M5	J	cytoplasmic	PROBABLE 30S RIBOSOMAL PROTEIN S12	14	14
rpsG	SMC01313	CFlFnPpPSSyO	M5	J	periplasmic	PROBABLE 30S RIBOSOMAL PROTEIN S7	17	23
rplQ	SMC01283	CFlFnPpPSSyO	M5	J	-	PROBABLE 50S RIBOSOMAL PROTEIN L17	16	21
rplJ	SMC01319	CFlFnPpPSSyO	M5	J	-	PROBABLE 50S RIBOSOMAL PROTEIN L10 (L8)	13	13
rpmD	SMC01291	FnPSO	M5	J	cytoplasmic	PROBABLE 50S RIBOSOMAL PROTEIN L30	1	1
rpsH	SMC01295	CFlFnPpPSSyO	M5	J	-	PROBABLE 30S RIBOSOMAL PROTEIN S8	19	24
nusG	SMC01322	CFlFnPpPSSyO	M5	K	cytoplasmic	PROBABLE TRANSCRIPTION ANTITERMINATION PROTEIN	8	17
rpoA	SMC01285	CFlFnPpPSSyO	M5	K	-	PROBABLE DNA-DIRECTED RNA POLYMERASE ALPHA CHAIN PROTEIN	15	25
fusA2	SMB20049	CFlFnPpPSSyO	M5	-	-	Putative elongation factor G protein.	4	5
infA	SMC02310	CFlFnPpPSSyO	M5	J	cytoplasmic	PROBABLE TRANSLATION INITIATION FACTOR IF-1 PROTEIN	9	12
Q92X26	SMB20136	CFlFnPpPSSyO	M45	-	-	Hypothetical protein SMb20136.	4	4
hpn8	SMA1288	CFlFnPpPSSyO	M45	-	-	Hypothetical protein hpn8.	5	5
Q92XP6	SMA2221	Fn	M45	-	periplasmic	Hypothetical protein.	1	1
Q92XP9	SMA2215	CFlFnPpPSSyO	M45	-	-	Putative GntR-family transcriptional regulator.	1	1
Q92XP7	SMA2219	CFlFnPpPSSyO	M45	-	periplasmic	Probable decarboxylase.	4	4
Q92X27	SMB20135	CFlFnPpPSSyO	M45	-	-	Putative 3-octaprenyl-4-hydroxybenzoate carboxy-lyase protein (EC 4.1.1.-).	3	3
Q92XP8	SMA2217	CFlFnPpPSSyO	M45	-	periplasmic	Putative decarboxylase.	5	5
hpn7	SMA1285	CFlFnPpPSSyO	M45	-	periplasmic	Probable decarboxylase (Hypthetical protein).	3	3
Q92U55	SMB21458	CFlFnPpPSSyO	M4	-	-	Putative sugar uptake ABC transporter permease protein.	2	18
aglK	SMC03065	CFlFnPpPSSyO	M4	G	-	ALPHA-GLUCOSIDES TRANSPORT ATP-BINDING ABC TRANSPORTER PROTEIN	10	26
Q92LJ0	SMC02473	CFlFnPpPSSyO	M4	G	periplasmic	PUTATIVE TRANSPORT SYSTEM PERMEASE ABC TRANSPORTER PROTEIN	10	32
Q92TH4	SMB20632	CFlFnPpPSSyO	M4	-	-	Putative sugar uptake ABC transporter permease protein.	11	33
Q92YW8	SMA1362	CFlFnPpPSSyO	M4	-	periplasmic	Putative inner-membrane permease.	22	58
Q92WV5	SMB20233	CFlFnPpPSSyO	M4	-	periplasmic	Putative sugar ABC transporter permease protein.	14	43
Q92MU6	SMC01979	CFlFnPpPSSyO	M4	G	periplasmic	PUTATIVE SUGAR TRANSPORT SYSTEM PERMEASE ABC TRANSPORTER PROTEIN	6	30
Q92WV7	SMB20231	CFlFnPpPSSyO	M4	-	-	Putative ABC transporter sugar-binding protein.	3	7
Q92WV3	SMB20235	CFlFnPpPSSyO	M4	-	periplasmic	Putative sugar ABC transporter ATP-binding protein.	14	28
Q92P67	SMC04256	CFlFnPpPSSyO	M4	G	-	PUTATIVE ATP-BINDING ABC TRANSPORTER PROTEIN	8	20
Q926F8	SMB20971	CFlFnPpPSSyO	M4	-	periplasmic	Putative sugar uptake ABC transporter periplasmic solute-binding protein.	8	26
thuK	SMB20328	CFlFnPpPSSyO	M4	-	periplasmic	ThuK (Probable trehalosemaltose transporter ATP-binding protein).	6	17
Q92YX9	SMA1341	CFlFnPpPSSyO	M4	-	periplasmic	Probable ABC transporter, permease protein.	11	43
lacK1	SMB20002	CFlFnPpPSSyO	M4	-	-	Probable lactose transport ATP-binding protein.	11	25
Q92VL6	SMB21106	CFlFnPpPSSyO	M4	-	-	Putative sugar uptake ABC transporter ATP-binding protein.	9	20
Q92TX6	SMB20661	CFlFnPpPSSyO	M4	-	-	Putative sugar uptake ABC transporter ATP-binding protein.	14	28
Q92V93	SMB21150	CFlFnPpPSSyO	M4	-	-	Putative sugar uptake ABC transporter permease protein.	3	25
gst9	SMC04141	FnPO	M4	O	periplasmic	PUTATIVE GLUTATHIONE S-TRANSFERASE PROTEIN	1	1
Q92LJ1	SMC02474	CFlFnPpPSSyO	M4	G	periplasmic	PUTATIVE ATP-BINDING ABC TRANSPORTER PROTEIN	14	30
Q92V92	SMB21151	CFlFnPpPSSyO	M4	-	periplasmic	Putative periplasmic solute-binding protein of a sugar uptake ABC transporter system.	1	2
Q92VI0	SMB21219	CFlFnPpPSSyO	M4	-	periplasmic	Putative sugar uptake ABC transporter permease protein.	7	24
Q92UN0	SMB20969	CFlFnPpPSSyO	M4	-	periplasmic	Putative sugar uptake ABC transporter permease protein.	9	31
Q92VI3	SMB21216	CFlFnPpPSSyO	M4	-	-	Putative sugar uptake ABC transporter ATP-binding protein.	8	21
Q92ZS2	SMA0711	CFlFnPpPSSyO	M4	-	periplasmic	Putative ABC transporter permease protein, MalFG family.	12	42
lacG	SMB21654	CFlFnPpPSSyO	M4	-	periplasmic	Probable lactose uptake ABC transporter permease protein.	2	12
Q92UQ2	SMB21602	CFlFnPpPSSyO	M4	-	periplasmic	Putative sugar uptake ABC transporter permease protein.	6	24
Q92XK1	SMA2309	CFlFnPpPSSyO	M4	-	periplasmic	Putative ABC transporter, permease.	11	35
Q92T65	SMC04137	CFlFnPpPSSyO	M4	G	periplasmic	PUTATIVE TRANSPORT SYSTEM PERMEASE ABC TRANSPORTER PROTEIN	7	26
Q92VL7	SMB21105	CFlFnPpPSSyO	M4	-	-	Putative sugar uptake ABC transporter permease protein.	7	23
Q92MU5	SMC01980	CFlFnPpPSSyO	M4	G	-	PUTATIVE SUGAR TRANSPORT SYSTEM ATP-BINDING ABC TRANSPORTER PROTEIN	12	26
Q92T68	SMC04140	CFlFnPpPSSyO	M4	G	-	PUTATIVE ATP-BINDING ABC TRANSPORTER PROTEIN	8	20
Q92TX8	SMB20658	CFlFnPpPSSyO	M4	-	-	Putative sugar uptake ABC transporter permease protein.	2	16
chvG	SMC04446	CFlFnPpPSSyO	M38	T	periplasmic	TRANSMEMBRANE HISTIDINE KINASE SENSORY PROTEIN (EC2.7.3.-)	6	48
nolF	SMA0876	CFlFnPpPSSyO	M334	-	-	Nodulation protein nolF.	1	1
nodN	SMA0874	CFnPpPSSyO	M334	-	-	PUTATIVE NODULATION PROTEIN	1	1
nolG	SMA0875	CFlFnPpPSSyO	M334	-	periplasmic	Nodulation protein nolG precursor.	3	3
paaG	SMB21633	CFlFnPpPSSyO	M33	-	periplasmic	Putative enoyl-CoA hydratase protein (EC 4.2.1.17).	1	1
Q92TG8	SMB21632	CFnPpPSSyO	M33	-	periplasmic	Putative 3-hydroxyacyl-CoA dehydrogenase protein (EC 1.1.1.35).	4	6
hbdA	SMC00727	CFnPpPSSyO	M33	I	periplasmic	PROBABLE 3-HYDROXYBUTYRYL-COA DEHYDROGENASE PROTEIN	6	12
Q92YU8	SMA1398	CFlFnPpPSSyO	M33	-	periplasmic	Putative.	1	1
Q92KZ6	SMC04397	CFlFnPpPSSyO	M33	C	periplasmic	PUTATIVE L-SORBOSONE DEHYDROGENASE, NADP DEPENDENT PROTEIN	1	1
Q92NC7	SMC01669	CFlFnPpPSSyO	M33	I	periplasmic	PUTATIVE ENOYL-COA HYDRATASE PROTEIN	2	2
Q92YU7	SMA1400	CFlFnPpPSSyO	M33	-	periplasmic	Probable fatty acid acyl-CoA.	8	11
Q92NF4	SMC01639	CFlFnPpPSSyO	M33	I	-	PUTATIVE ACYL-COA DEHYDROGENASE PROTEIN	6	9
Q92KZ5	SMC04398	CFnPpPSSyO	M33	I	-	PUTATIVE ENOYL-COA HYDRATASE PROTEIN	5	6
Q930K4	SMA0355	CFlFnPpPSSyO	M329	-	-	Putative LysR-type regulator.	2	2
Q930K5	SMA0353	CFlFnPpPSSyO	M329	-	periplasmic	Putative LysR-type regulator.	1	3
Q930K3	SMA0356	*	M329	-	periplasmic	Hypothetical protein.	1	1
Q930B0	SMA0552	CFlFnPpPSSyO	M327	-	-	Allantoinase, putative.	2	2
Q930A9	SMA0554	*	M327	-	periplasmic	Hypothetical protein.	1	1
Q930B1	SMA0551	CFlFnPpPSSyO	M327	-	-	Hydrolase, putative.	1	1
Q92ZA0	SMA1091	*	M322	-	periplasmic	Hypothetical protein SMa1091.	1	1
Q92ZA1	SMA1089	*	M322	-	periplasmic	Hypothetical protein SMa1089.	2	2
Q92ZA2	SMA1087	CFlFnPpPSSyO	M322	-	periplasmic	Putative cation transport P-type ATPase.	1	1
Q92Z93	SMA1101	*	M321	-	periplasmic	Hypothetical protein.	2	2
Q92Z94	SMA1100	C	M321	-	periplasmic	Hypothetical protein.	1	1
Q92Z92	SMA1103	CFlFnPpPSSyO	M321	-	-	Probable adenylate cyclase (EC 4.6.1.1).	1	1
fixS	SMA1208	FnPS	M320	-	periplasmic	Nitrogen fixation protein fixS.	1	1
fixI	SMA1209	CFlFnPpPSSyO	M320	-	periplasmic	Nitrogen fixation protein fixI (E1-E2 type cation ATPase fixI) (EC 3.6.3.-).	2	2
fixH	SMA1210	FnPpPO	M320	-	periplasmic	Nitrogen fixation protein fixH.	2	2
Q92YL7	SMA1576	CFnPpPSO	M319	-	periplasmic	Probable CpaB2 pilus assembly protein.	1	3

Q92YL9	SMA1572	*	M319	-	periplasmic	Hypothetical protein.	1	1
Q92YL8	SMA1573	CFnPpPSSyO	M319	-	-	Probable CpaE2 pilus assembly protein.	2	2
ndiA-2	SMB20228	*	M313	-	-	Putative nutrient deprivation-induced protein.	2	2
ndiA-1	SMB20227	FnS	M313	-	periplasmic	Probable nutrient deprivation-induced protein.	1	1
ndiB	SMB20229	*	M313	-	periplasmic	NdiB (Probable nutrient deprivation-induced protein).	1	1
Q92WU7	SMB20242	*	M312	-	periplasmic	Hypothetical protein SMb20242.	1	1
Q92WU9	SMB20240	*	M312	-	periplasmic	Hypothetical protein SMb20240.	1	1
Q92WU8	SMB20241	*	M312	-	periplasmic	Hypothetical protein SMb20241.	2	2
expE2	SMB21313	CFlFnPpPSSyO	M306	-	-	PUTATIVE BIFUNCTIONAL GLYCOSYLTRANSFERASE, FORMING ALPHA-GLYCO:	4	7
expE3	SMB21312	CPSSyO	M306	-	-	PUTATIVE METHYLTRANSFERASE	2	2
expE4	SMB21311	CFlFnPpPSSyO	M306	-	-	PUTATIVE GLYCOSYLTRANSFERASE, FORMING ALPHA-GYLCOSYL LINKAGES	5	8
lpsE	SMC01220	CFlFnPpPSSyO	M299	M	-	PUTATIVE LIPOPOLYSACCHARIDE CORE BIOSYNTHESIS GLYCOSYL TRANSFERASE PROTEIN	1	1
lpsD	SMC01221	CFlFnPpPSSyO	M299	M	-	PUTATIVE LIPOPOLYSACCHARIDE CORE BIOSYNTHESIS GLYCOSYL TRANSFERASE PROTEIN	2	2
lpsC	SMC01222	CFlFnPpPSSyO	M299	M	periplasmic	PUTATIVE LIPOPOLYSACCHARIDE CORE BIOSYNTHESIS GLYCOSYL TRANSFERASE PROTEIN	1	1
nuoN	SMC01927	CFlFnPpPSSyO	M29	C	periplasmic	PROBABLE NADH DEHYDROGENASE I CHAIN N TRANSMEMBRANE PROTEIN	6	7
nuoK1	SMC01924	CFlFnPpPSSyO	M29	C	periplasmic	PROBABLE NADH DEHYDROGENASE I CHAIN K TRANSMEMBRANE PROTEIN	5	5
nuoJ	SMC01923	CFnPpPSSyO	M29	C	periplasmic	PROBABLE NADH DEHYDROGENASE I CHAIN J TRANSMEMBRANE PROTEIN	6	6
nuoM	SMC01926	CFlFnPpPSSyO	M29	C	periplasmic	PROBABLE NADH DEHYDROGENASE I CHAIN M TRANSMEMBRANE PROTEIN	5	5
Q92YN3	SMA1541	CFlFnPpPSSyO	M29	-	periplasmic	Putative oxidoreductase (EC 1.6.5.3).	2	2
Q92YN5	SMA1538	CFlFnPpPSSyO	M29	-	periplasmic	Putative oxidoreductase.	5	5
Q92YN6	SMA1536	CFlFnPpPSSyO	M29	-	periplasmic	NuoM2 NADH-Ubiquinone/plastoquinone (Complex I) oxidoreductase (EC 1.6.5.3).	5	5
nuoG1	SMC01920	CFlFnPpPSSyO	M29	C	periplasmic	PROBABLE NADH DEHYDROGENASE I CHAIN G PROTEIN	2	2
nuoL	SMC01925	CFlFnPpPSSyO	M29	C	periplasmic	PROBABLE NADH DEHYDROGENASE I CHAIN L TRANSMEMBRANE PROTEIN	7	7
msbA1	SMB20813	CFlFnPpPSSyO	M285	-	periplasmic	Putative lipid A + LPS core exporting ABC transporter protein, consisting of ATP-binding and permease domains.	2	2
ndvA	SMC03900	CFlFnPpPSSyO	M285	V	periplasmic	BETA-1-->2GLUCAN EXPORT ATP-BINDING PROTEIN	4	4
exsA	SMB20941	CFlFnPpPSSyO	M285	-	periplasmic	ExsA (MsbA-like saccharide exporting ABC transporter protein, consisting of ATP-binding and permease domains).	2	2
Q930Y4	SMA0117	Fn	M275	-	periplasmic	Hypothetical protein.	1	1
Q930Y5	SMA0116	CFlFnPpPSSyO	M275	-	-	Putative DnaJ/CbpA-type protein.	2	3
Q92R78	SMC02376	CFlFnPpPSSyO	M275	O	periplasmic	PUTATIVE HEAT SHOCK PROTEIN	1	1
Q92VP9	SMB21071	CFlFnPpPSSyO	M266	-	periplasmic	Putative initiating glycosyltransferase, transferring a sugar residue to undecaprenolphosphate protein.	1	1
Q92QS4	SMC01794	CFlFnPpPSSyO	M266	M	periplasmic	PUTATIVE POLYSACCHARIDE EXPORT SYSTEM PERIPLASMIC TRANSMEMBRANE PROTEIN	1	1
Q92QS6	SMC01792	CFlFnPpPSSyO	M266	M	-	PUTATIVE SUGAR TRANSFERASE PROTEIN	3	5
Q930L4	SMA0337	*	M263	-	-	Hypothetical protein.	1	1
Q930L5	SMA0335	CFlFnPpPSSyO	M263	-	periplasmic	Putative.	2	3
Q930L3	SMA0339	CFlFnPpPSSyO	M263	-	-	Putative.	1	1
Q92P54	SMC04273	CFlFnPpPSSyO	M26	I	-	PUTATIVE 3-OXOACYL-ACYL-CARRIER-PROTEIN SYNTHASE	3	3
fabG	SMC00572	CFlFnPpPSSyO	M26	Q	periplasmic	PROBABLE 3-OXOACYL-ACYL-CARRIER PROTEIN REDUCTASE	2	2
nodE	SMA0853	CFlFnPpPSSyO	M26	-	-	Nodulation protein E (Host-specificity of nodulation protein B) (EC 2.3.1.-).	3	3
fabF	SMC00574	CFlFnPpPSSyO	M26	I	-	PROBABLE 3-OXOACYL-ACYL-CARRIER-PROTEIN SYNTHASE II	2	2
nodG	SMA0854	CFlFnPpPSSyO	M26	-	periplasmic	Nodulation protein G (Host-specificity of nodulation protein C).	5	5
Q92P52	SMC04277	CFnPpPSSyO	M26	I	-	PUTATIVE (3R)-HYDROXYMYRISTOYL-[ACYL CARRIER] DEHYDRATASE TRANSMEMBRANE PROTEIN	1	1
fabD	SMC00571	CFlFnPpPSSyO	M26	I	periplasmic	PROBABLE MALONYL COA-ACYL CARRIER PROTEIN TRANSACYLASE	6	8
acpP	SMC00573	CFlFnPpPSSyO	M26	I	periplasmic	ACYL CARRIER PROTEIN	7	7
phbB	SMC03878	CFlFnPpPSSyO	M26	Q	periplasmic	ACETOACETYL-COA REDUCTASE PROTEIN	1	1
Q92P53	SMC04275	CFnPpPS	M26	I	periplasmic	PUTATIVE ACYL-ACP SYNTHASE PROTEIN	2	2
plsX	SMC01784	CFlFnPpPSSyO	M26	I	-	PUTATIVE FATTY ACID/PHOSPHOLIPID SYNTHESIS PROTEIN	2	2
Q92WY1	SMB20181	Fn	M254	-	periplasmic	Putative ABC transporter periplasmic solute-binding protein.	1	1
Q92WX9	SMB20183	CFlFnPpPSSyO	M254	-	-	Putative ABC transporter ATP-binding protein.	1	2
Q92WY0	SMB20182	CFlFnPpPSSyO	M254	-	periplasmic	Putative ABC transporter protein.	2	2
nosD	SMA1183	CFnPpPSSyO	M248	-	periplasmic	NosD protein (NosD periplasmic copper-binding).	4	4
nosZ	SMA1182	CFnPpPSSyO	M248	-	periplasmic	Nitrous-oxide reductase precursor (EC 1.7.99.6) (N(2)OR) (N2O reductase).	2	2
nosR	SMA1179	CFlFnPpPSSyO	M248	-	periplasmic	NosR Regulatory protein for N2O reductase.	2	2
nodB	SMA0868	CFlFnPpPSSyO	M245	-	-	NODB CHITOOLIGOSACCHARIDE DEACETYLAS (putative nodulation protein)	2	2
nodA	SMA0869	Fn	M245	-	-	Nodulation protein A (EC 2.3.1.-).	1	1
nodC	SMA0866	CFnPpPSSyO	M245	-	periplasmic	N-acetylglucosaminyltransferase (EC 2.4.1.-) (Nodulation protein C).	1	1
fixO3	SMA0615	CFnPpPSSyO	M24	-	periplasmic	FixO3 cytochrome-c oxidase subunit.	7	7
fixN	SMA1220	CFlFnPpPSSyO	M24	-	periplasmic	Cytochrome c oxidase subunit 1 homolog, bacteroid (EC 1.9.3.1) (Cytochrome c oxidase polypeptide I homolog) (Cytochrome	7	7
fixP	SMA1213	CFnPpPSSyO	M24	-	-	FixP (FixP1 Di-heme cytochrome c).	7	7
fixG	SMA1211	CFnPpPSSyO	M24	-	-	Nitrogen fixation protein fixG.	10	10
fixQ3	SMA0616	*	M24	-	cytoplasmic	FixQ3 nitrogen fixation protein.	1	1
fixN3	SMA0612	CFlFnPpPSSyO	M24	-	periplasmic	FixN3 cytochrome c oxidase subunit 1.	7	7
fixN2	SMA0765	CFlFnPpPSSyO	M24	-	periplasmic	FixN2 cytochrome c oxidase polypeptide I (EC 1.9.3.1).	7	7
fixO2	SMA0766	CFnPpPSSyO	M24	-	-	FixO2 cytochrome c oxidase (EC 1.9.3.1).	7	7
fixP3	SMA0617	CFnPpPSSyO	M24	-	periplasmic	FixP3 cytochrome c oxidase membrane anchored subunit.	8	8
fixP2	SMA0769	CFnPpPSSyO	M24	-	-	FixP2 cytochrome c oxidase (EC 1.9.3.1).	7	7
fixO	SMA1216	CFnPpPSSyO	M24	-	-	FixO (FixO1 c-type cytochrome).	7	7
narB	SMB20986	CFlFnPpPSSyO	M238	-	-	Putative nitrate reductase, large subunit protein (EC 1.7.99.4)	2	12
nirB	SMB20984	CFlFnPpPSSyO	M238	-	-	PUTATIVE NITRITE REDUCTASE [NAD(EC1.6.6.4)	3	7
nirD	SMB20985	CFnPpPSO	M238	-	periplasmic	PUTATIVE NITRITE REDUCTASE [NAD(EC1.6.6.4)	1	2
Q92YF3	SMA1688	CFlFnPpPSSyO	M227	-	-	Putative two-component response regulator.	2	2
Q92YF4	SMA1686	CFlFnPpPSSyO	M227	-	-	Putative two-component response regulator.	2	3
Q92YF5	SMA1684	CFlFnPpPSSyO	M227	-	periplasmic	Putative two-component sensor kinase.	4	5
kpsF1	SMB20809	CFnPpPSSyO	M21	-	periplasmic	PUTATIVE PROTEIN INVOLVED IN ASSEMBLY, OPERATION OR REGULATION OF	3	19
kpsF2	SMB20830	CFnPpPSSyO	M21	-	periplasmic	PUTATIVE PROTEIN INVOLVED IN ASSEMBLY, OPERATION OR REGULATION OF	3	19
kpsF3	SMC02268	CFlFnPpPSSyO	M21	M	periplasmic	CAPSULE EXPRESSION PROTEIN	3	19
Q92VH5	SMB21225	CFlFnPpPSSyO	M208	-	periplasmic	Putative inositol monophosphatase, possibly involved in PAPS metabolism protein.	1	1
nodP2	SMB21223	CFnPpPSSyO	M208	-	-	Putative sulfate adenylyltransferase subunit 2 protein (EC 2.7.7.4).	4	4
nodQ2	SMB21224	CFlFnPpPSSyO	M208	-	-	Putative sulfate adenylyltransferase subunit 1 adenylsulfate kinase protein (EC 2.7.1.25).	5	5
**dctQ**	SMB21352	CFnPpPSSyO	M20	-	periplasmic	PUTATIVE C4-DICARBOXYLATE SMALL MEMBRANE TRANSPORT PROTEIN	11	22
**dctP**	SMB21353	CFlFnPpPSSyO	M20	-	periplasmic	PUTATIVE PERIPLASMIC C4-DICARBOXYLATE TRANSPORT PROTEIN	13	29
rkpR/kpsE	SMB20831	CPSO	M154	-	periplasmic	PUTATIVE POLYSACCHARIDE EXPORT-ASSOIATED PROTEIN	6	6
rkpS	SMB20832	CFlFnPpPSSyO	M154	-	-	PUTATIVE CELL SURFACE POLYSACCHARIDE EXPORT ABC-2 TRANSPORTER A	6	8
rkpT1	SMB20833	CPSSyO	M154	-	-	PUTATIVE CELL SURFACE POLYSACCHARIDE EXPORT ABC-2 TRANSPORTER P	5	5
rkpZ1	SMB20834	CPO	M154	-	-	PROBABLE SURFACE SACCHARIDE SYNTHESIS PROTEIN, POSSIBLY INVOLVED	6	7
norQ	SMA1272	CFlFnPpPSSyO	M153	-	periplasmic	NorQ protein required for nitric oxide reductase activity (EC 1.7.99.7) (Putative chaperone).	3	3
norC	SMA1276	FnPpPSSy	M153	-	periplasmic	NorC nitric oxide reductase (EC 1.7.99.7) (Nitric oxide reductase small subunit).	2	2
norB	SMA1273	CFnPpPSSyO	M153	-	periplasmic	NorB nitric oxide reductase (EC 1.7.99.7) (Nitric oxide reductase large subunit).	3	3
norD	SMA1269	FnPpPSSyO	M153	-	periplasmic	NorD protein required for nitric oxide reductase (Nor) activity (EC 1.7.99.7).	2	2
nosX	SMA1188	CFnPpPSSyO	M151	-	periplasmic	NosX protein required for nitrous oxide reduction.	1	1
nosL	SMA1186	CFnPpPSSy	M151	-	periplasmic	NosL protein required for nitrous oxide reduction.	4	4
nosY	SMA1185	CFnPpPSSyO	M151	-	periplasmic	NosY nitrous oxide metabolic protein.	2	2
nosF	SMA1184	CFlFnPpPSSyO	M151	-	-	NosF protein.	3	3
livH	SMC01951	CFlFnPpPSSyO	M15	E	periplasmic	PROBABLE HIGH-AFFINITY BRANCHED-CHAIN AMINO ACID TRANSPORT PERMEASE ABC TRANSPORTER PRO	14	15
livJ	SMC00078	CFlFnPpPSSyO	M15	E	periplasmic	PROBABLE LEU/ILE/VAL-BINDING PROTEIN	12	12
Q92ZZ6	SMA0576	CFlFnPpPSSyO	M15	-	periplasmic	Leu or Leu/Val/Ile Transport Binding Protein.	12	14
Q92TN4	SMB20784	CFlFnPpPSSyO	M15	-	periplasmic	Putative branched-chain amino acid uptake ABC transporter ATP-binding protein.	8	11
livG	SMC01949	CFlFnPpPSSyO	M15	E	periplasmic	PROBABLE HIGH-AFFINITY BRANCHED-CHAIN AMINO ACID TRANSPORT ATP-BINDING ABC TRANSPORTER PR	12	13
Q92MN0	SMC02356	CFlFnPpPSSyO	M15	E	periplasmic	PUTATIVE BRANCHED CHAIN AMINO ACID BINDING PERIPLASMIC ABC TRANSPORTER PROTEIN	13	13
livM	SMC01950	CFlFnPpPSSyO	M15	E	periplasmic	PROBABLE HIGH-AFFINITY BRANCHED-CHAIN AMINO ACID TRANSPORT PERMEASE ABC TRANSPORTER PRO	15	17
Q92MM7	SMC02359	CFlFnPpPSSyO	M15	E	periplasmic	PUTATIVE HIGH-AFFINITY BRANCHED-CHAIN AMINO ACID TRANSPORT PERMEASE ABC TRANSPORTER PROT	14	17
Q92LQ5	SMC03118	CFlFnPpPSSyO	M15	E	periplasmic	PUTATIVE PERMEASE ABC TRANSPORTER PROTEIN	12	15
Q92LQ7	SMC03120	CFlFnPpPSSyO	M15	E	periplasmic	PUTATIVE ATP-BINDING ABC TRANSPORTER PROTEIN	12	13
Q92LQ4	SMC03117	CFnPpPSSyO	M15	E	-	PUTATIVE PERMEASE ABC TRANSPORTER PROTEIN	9	10
Q92MM9	SMC02357	CFlFnPpPSSyO	M15	E	periplasmic	PUTATIVE HIGH-AFFINITY BRANCHED-CHAIN AMINO ACID TRANSPORT ATP-BINDING ABC TRANSPORTER PR	9	10
Q92LQ6	SMC03119	CFlFnPpPSSyO	M15	E	periplasmic	PUTATIVE ATP-BINDING ABC TRANSPORTER PROTEIN	12	12
Q92LQ8	SMC03121	CFlFnPpPSSyO	M15	E	periplasmic	PUTATIVE PERIPLASMIC BINDING ABC TRANSPORTER PROTEIN	1	1
livK	SMC01946	CFlFnPpPSSyO	M15	E	periplasmic	PUTATIVE LEUCINE-SPECIFIC BINDING PROTEIN PRECURSOR	12	12
Q92TN3	SMB20785	CFlFnPpPSSyO	M15	-	periplasmic	Putative branched-chain amino acid uptake ABC transporter ATP-binding protein.	13	16
Q92MM8	SMC02358	CFlFnPpPSSyO	M15	E	-	PUTATIVE HIGH-AFFINITY BRANCHED-CHAIN AMINO ACID TRANSPORT ATP-BINDING ABC TRANSPORTER PR	11	12
livF	SMC01948	CFlFnPpPSSyO	M15	E	-	PROBABLE HIGH-AFFINITY BRANCHED-CHAIN AMINO ACID TRANSPORT ATP-BINDING ABC TRANSPORTER PR	10	11

tyv	SMB20244	CFlFnPpPSSyO	M148	-	-	Putative CDP-tyvelose-2-epimerase protein (EC 5.1.3.-).	4	4
rmlB	SMB20239	CFlFnPpPSSyO	M148	-	periplasmic	Putative dTDP-glucose 4,6-dehydratase protein (EC 4.2.1.46).	2	2
Q92WU6	SMB20243	*	M148	-	periplasmic	Putative glycosyltransferase protein.	1	1
Q92WU4	SMB20245	CFlFnPpPSSyO	M148	-	periplasmic	Putative NDP-glucose dehydrataseepimerase protein.	3	3
Q930W3	SMA0151	CFlFnPpPSSyO	M12	-	periplasmic	Hypothetical protein.	9	18
Q92WM5	SMB20321	CFlFnPpPSSyO	M12	-	periplasmic	Hypothetical protein SMb20321.	2	15
Q92UM2	SMB20979	CFlFnPpPSSyO	M12	-	periplasmic	Putative large C4-dicarboxylate uptake permease protein, DedA family protein.	3	17
Q92U72	SMB21437	CPpPSyO	M12	-	periplasmic	Putative C4-dicarboxylate transport system, permease small protein TRANSMEMBRANE.	1	2
Q92PN6	SMC00273	CFlFnPpPSSyO	M12	G	periplasmic	PUTATIVE TRANSMEMBRANE PROTEIN	2	16
Q92KC1	SMC00272	Fn	M12	G	periplasmic	HYPOTHETICAL TRANSMEMBRANE PROTEIN	1	2
Q92WI1	SMB20372	CFlFnPpPSSyO	M12	-	periplasmic	Hypothetical protein SMb20372.	3	17
Q92PP2	SMC00266	CFlFnPpPSSyO	M12	G	periplasmic	PUTATIVE TRANSMEMBRANE PROTEIN	4	16
Q92WM4	SMB20322	FnSO	M12	-	periplasmic	Hypothetical protein SMb20322.	1	1
Q92U73	SMB21436	CFlFnPpPSSyO	M12	-	periplasmic	Putative C4-dicarboxylate transport system, permease large protein TRANSMEMBRANE.	2	16
Q92UM1	SMB20980	CFnPpPSSyO	M12	-	-	Putative small C4-dicarboxylate uptake permease protein.	1	2
**dctM**	SMB21351	CFlFnPpPSSyO	M12	-	periplasmic	Putative C4-dicarboxylate large membrane transport protein.	1	15
Q92WB7	SMB20444	CFlFnPpPSSyO	M12	-	periplasmic	Hypothetical protein SMb20444.	4	17
Q92WI0	SMB20373	*	M12	-	periplasmic	Hypothetical protein SMb20373.	1	2
Q92WP2	SMB20297	CFlFnPpPSSyO	M12	-	periplasmic	Putative permease protein.	2	16
Q92WB6	SMB20445	CFlFnPpPSSyO	M12	-	-	Putative alcohol dehydrogenase protein.	1	1
Q92WP1	SMB20298	CFnPpPSSyO	M12	-	-	Hypothetical protein SMb20298.	1	1
Q92WB8	SMB20443	CFnPpPSO	M12	-	periplasmic	Putative permease protein.	1	2
fixX	SMA0816	FnPSSyO	M106	-	cytoplasmic	Ferredoxin-like protein.	2	2
fixA	SMA0822	CFnPpPSSyO	M106	-	-	Protein fixA.	4	4
fixB	SMA0819	CFnPpPSSyO	M106	-	-	Protein fixB.	8	8
fixC	SMA0817	CFlFnPpPSSyO	M106	-	periplasmic	Protein fixC.	3	3
Q92ZD6	SMA1018	*	--	-	periplasmic	Hypothetical protein.	1	1
ntrY	SMC01044	CFlFnPpPSSyO	--	T	periplasmic	TRANSMEMBRANE NITROGEN REGULATION PROTEIN (EC2.7.3.-)	4	16
ntrB	SMC01042	CFlFnPpPSSyO	--	T	periplasmic	NITROGEN REGULATION PROTEIN	8	8
cyoB	SMB21488	CFlFnPpPSSyO	--	-	-	PUTATIVE CYTOCHROME O UBIQUINOL OXIDASE CHAIN I (EC1.10.3.-)	6	13
exsH	SMB20932	CFlFnPpPSyO	--	-	periplasmic	ENDO-1,3-1,4-BETA-GLYCANASE, C-TERMINAL SECRETION SIGNAL	1	1
Q92ZU2	SMA0675	CFlFnPpPSSyO	--	-	-	Cation (Ca) exchange protein, possible.	1	1
exoK	SMB20955	CFnPpSO	--	-	periplasmic	Endo-1,3-1,4-beta-glycanase exoK precursor (EC 3.2.1.-) (Succinoglycan biosynthesis protein exoK).	2	2
Q92R71	SMC02383	*	--	M	periplasmic	HYPOTHETICAL TRANSMEMBRANE PROTEIN	1	1
expD2	SMB21315	CFlFnPpPSSyO	--	-	periplasmic	PUTATIVE PROTEIN SECRETION PROTEIN, HLYD FAMILY MEMBRANE FUSION P	2	8
Q92JY8	SMC00663	*	--	-	-	HYPOTHETICAL PROTEIN	1	1
exoB	SMB20942	CFlFnPpPSSyO	--	-	-	UDP-glucose 4-epimerase (EC 5.1.3.2) (Galactowaldenase).	3	3
syrA	SMA0838	*	--	-	periplasmic	SYRA PROTEIN INVOLVED IN EPS PRODUCTION	1	3
Q92ZA5	SMA1082	Fn	--	-	-	Hypothetical protein SMa1082.	1	1
Q92WK0	SMB20353	CFlFnPpPSSyO	--	-	periplasmic	Putative oxidoreductase protein.	1	1
fabB	SMC00327	CFlFnPpPSSyO	--	I	periplasmic	PROBABLE 3-OXOACYL-ACYL-CARRIER-PROTEIN SYNTHASE I	1	3
Q92UL3	SMB20989	CFlFnPpPSSyO	--	-	periplasmic	PUTATIVE STOMATIN-LIKE PROTEIN	1	2
Q92UD1	SMB20907	*	--	-	periplasmic	Hypothetical protein SMb20907.	1	1
exoX	SMB20947	Pp	--	-	periplasmic	Exopolysaccharide production repressor protein	4	4
expA1	SMB21319	*	--	-	-	PUTATIVE MEMBRANE-ANCHORED PROTEIN	1	1
rkpI	SMC02270	CFnPpPSSy	--	M	periplasmic	CAPSULAR POLYSACCHARIDE BIOSYNTHESIS\EXPORT TRANSMEMBRANE PROTEIN	2	2
Q930C2	SMA0523	*	--	-	-	Hypothetical protein.	1	2
Q92XM0	SMA2273	*	--	-	-	Hypothetical protein.	1	1
exoW	SMB21690	CFlFnPpPSSyO	--	-	periplasmic	Succinoglycan biosynthesis protein exoW (EC 2.-.-.-).	1	1
Q92N90	SMC01580	*	--	-	periplasmic	HYPOTHETICAL TRANSMEMBRANE PROTEIN	1	1
Q92TQ5	SMB20753	CFlFnPpPSSyO	--	-	-	Putative acyl-CoA dehydrogenase protein (EC 1.3.99.-).	1	5
galM	SMC03798	CFnPpPSSyO	--	G	-	ALDOSE 1-EPIMERASE (EC5.1.3.3)	2	7
Q930V8	SMA0163	CFlFnPpPSSyO	--	-	-	Probable PilQ pilus assembly protein.	1	2
exoU	SMB20948	FnPp	--	-	periplasmic	Succinoglycan biosynthesis protein exoU (EC 2.-.-.-)	9	11
Q92VU4	SMB21031	*	--	-	-	Hypothetical membrane-anchored protein.	1	1
Q92YE6	SMA1700	*	--	-	periplasmic	Hypothetical protein.	1	1
**dctD**	SMB20613	CFlFnPpPSSyO	--	-	periplasmic	C4-dicarboxylate transport transcriptional regulatory protein dctD.	1	2
nodD2	SMA0757	CFlFnPpPSSyO	--	-	periplasmic	NODD2 NOD BOX-DEPENDENT TRANSCRIPTION ACTIVATOR	3	6
rlpA	SMC01187	CFlFnPpPSSyO	--	M	periplasmic	RARE LIPOPROTEIN A PRECURSOR	1	15
lepB	SMC02653	CFlFnPpPSSyO	--	U	periplasmic	TRANSMEMBRANE SIGNAL PEPTIDASE I (EC3.4.21.89)	1	16
pphA	SMB21213	CFlFnPpPSSyO	--	-	periplasmic	Putative serinethreonine protein phosphatase (EC 3.1.3.16).	1	1
Q92N89	SMC01581	*	--	-	periplasmic	HYPOTHETICAL TRANSMEMBRANE PROTEIN	1	1
Q92XL9	SMA2275	*	--	-	periplasmic	Hypothetical protein.	1	1
nfeD	SMB20990	CFnPpPSSyO	--	-	periplasmic	PROBABLE MEMBRANE PROTEIN NECESSARY FOR NODULATION COMPETITIVE	1	1
fixL	SMA1142	CFlFnPpPSSyO	--	-	periplasmic	FixL-related histidine kinase.	1	1
exoR	SMC02078	FnPpPSSyO	--	R	periplasmic	EXOPOLYSACCHARIDE BIOSYNTHESIS REGULATORY PROTEIN	2	6
Q92ZC3	SMA1046	CFlFnPpPSSyO	--	-	periplasmic	Probable adenylate cyclase (EC 4.6.1.1).	1	4
Q92Z75	SMA1141	CFnPpPSSyO	--	-	periplasmic	Putative fnr/crp family transcriptional regulator.	1	1
PHK2_RHIME	SMA1084	CFlFnPpPSSyO	--	-	-	Probable phosphoketolase 2 (EC 4.1.2.-).	1	1
Q92U36	SMB21480	*	--	-	periplasmic	Hypothetical protein SMb21480.	1	1
nodF	SMA0852	Fn	--	-	periplasmic	NODF ACYL CARRIER PROTEIN	1	1
napA	SMA1236	CFlFnPpPSSyO	--	-	periplasmic	--	3	18
Q92ZW7	SMA0630	CFlFnPpPSSyO	--	-	periplasmic	Conserved hypothetical membrane protein.	1	1
hupB	SMC01906	CFlFnPpPSSyO	--	L	periplasmic	HISTONE-LIKE PROTEIN	4	4
Q92VQ7	SMB21063	*	--	-	-	Hypothetical nucleotide-binding protein.	1	1
**mucR**	SMC00058	FnPpPSO	--	K	periplasmic	TRANSCRIPTION REGULATOR PROTEIN	3	28
ihfA	SMC01786	CFlFnPpPSSyO	--	L	-	INTEGRATION HOST FACTOR ALPHA-SUBUNIT	1	24
Q92XM4	SMA2265	*	--	-	cytoplasmic	Hypothetical protein.	1	1
Q92ZY8	SMA0592	CFnPpPSSyO	--	-	-	Hypothetical protein.	1	1
Q92V56	SMB21195	*	--	-	periplasmic	Hypothetical protein SMb21195.	1	1
fixT	SMA1226	*	--	-	periplasmic	Transcriptional regulator protein fixT (Antikinase fixT).	1	1
Q92PQ4	SMC04437	*	--	-	periplasmic	HYPOTHETICAL PROTEIN	1	1
nodH	SMA0851	*	--	-	periplasmic	Nodulation protein H (EC 2.8.2.-) (Host-specificity of nodulation protein D)	1	2
exoQ	SMB21505	FlFnPpPO	--	-	-	Putative membrane protein, similar to Wzy-like polysaccharide polymerase ExoQ.	1	1
Q92U46	SMB21470	*	--	-	periplasmic	Hypothetical protein SMb21470.	1	1
mocF	SMB20820	CFlFnPpPSSyO	--	-	periplasmic	PUTATIVE FERREDOXIN REDUCTASE	1	5
Q92VQ6	SMB21064	*	--	-	cytoplasmic	Hypothetical protein SMb21064.	1	1
TRm30.3	SMC02301	*	--	-	periplasmic	PUTATIVE TRANSPOSASE NUMBER 3 FOR INSERTION SEQUENCE ISRM30	1	2
Q92JU8	SMC02493	*	--	-	-	HYPOTHETICAL PROTEIN	1	1
mocD	SMB20818	CFnPpSO	--	-	periplasmic	PUTATIVE HYDROCARBON OXYGENASE	2	14
xthA1	SMC00956	CFlFnPpPSSyO	--	L	-	PROBABLE EXODEOXYRIBONUCLEASE III PROTEIN	1	1
cysG	SMC01053	CFlFnPpPSSyO	--	H	periplasmic	SIROHEME SYNTHASE (EC2.1.1.107)	1	9
Q92ZW3	SMA0637	*	--	-	periplasmic	Hypothetical protein.	1	1
nuoK2	SMA1544	*	--	-	periplasmic	Putative NADH-ubiquinone oxidoreductase subunit (NuoK2 NADH I chain K) (EC 1.6.5.3).	2	2
expA6	SMB21323	*	--	-	periplasmic	PUTATIVE PROTEIN, PROBABLY EXPORTED TO PERIPLASMA	2	2
ropB2	SMC00257	FnPpPS	--	M	periplasmic	OUTER-MEMBRANE PROTEIN	1	2
nodD3	SMA0840	CFlFnPpPSSyO	--	-	periplasmic	NODD3 TRANSCRIPTIONAL REGULATOR	3	11
napD	SMA1239	PpP	--	-	periplasmic	NAPD COMPONENT OF PERIPLASMIC NITRATE REDUCTASE	1	1
exoZ	SMB20943	CFlFnPpPSSyO	--	-	periplasmic	Exopolysaccharide production protein exoZ	2	2
ihfB	SMC01134	CFlFnPpPSSyO	--	L	periplasmic	INTEGRATION HOST FACTOR BETA-SUBUNIT	1	6
exoO	SMB20959	CFnPpSO	--	-	periplasmic	Succinoglycan biosynthesis protein exoO (EC 2.-.-.-).	2	2
exsG	SMB20933	CFlFnPpPSSyO	--	-	periplasmic	Putative two-component sensor histidine kinase protein.	1	2
nifR33	SMC01041	CFlFnPpPSSyO	--	J	-	PUTATIVE NIFR3-LIKE PROTEIN	1	1
nodQ	SMA0857	CFlFnPpPSSyO	--	-	-	NodQ bifunctional enzyme (Nodulation protein Q) [Includes: Sulfate adenylyltransferase subunit 1 (EC 2.7.7.4) (Sulfate adenyl	6	6
Q92ZU3	SMA0674	*	--	-	periplasmic	Hypothetical protein.	1	1
Q92VU5	SMB21030	*	--	-	periplasmic	Hypothetical protein SMb21030.	1	1
nodI	SMA0864	CFlFnPpPSSyO	--	-	-	NODI MEMBRANE TRANSPORT PROTEIN	5	40
Q92PQ3	SMC00255	*	--	-	periplasmic	HYPOTHETICAL PROTEIN	1	1
**rpoN**	SMC01139	CFnPpPSSyO	--	K	periplasmic	RNA POLYMERASE SIGMA-54 FACTOR	10	16
ctaE	SMC00013	CFlFnPpPSSyO	--	C	periplasmic	TRANSMEMBRANE CYTOCHROME C OXIDASE SUBUNIT III (EC1.9.3.1)	4	16

Q92XM5	SMA2263	*	--	-	cytoplasmic	Hypothetical protein.	1	1
Q930V7	SMA0164	*	--	-	periplasmic	Hypothetical protein.	1	1
nodM	SMA0878	CFlFnPpPSSyO	--	-	-	NODM GLUTAMINE AMINOTRANSFERASE	2	4
Q92UC2	SMB21682	*	--	-	cytoplasmic	Hypothetical protein SMb21682.	1	1
Q92PT0	SMC00958	*	--	-	periplasmic	HYPOTHETICAL/UNKNOWN PROTEIN	1	1
rkpH	SMC02271	CFlFnPpPSSyO	--	Q	periplasmic	RIBITOL TYPE DEHYDROGENASE PROTEIN	1	4
exoM	SMB20958	CFlFnPpPSSyO	--	-	-	Succinoglycan biosynthesis protein exoM (EC 2.-.-.-).	2	2
Q92YE7	SMA1699	*	--	-	periplasmic	Hypothetical protein.	1	1
Q92YE5	SMA1706	*	--	-	periplasmic	Hypothetical protein.	1	1
Q92ZW2	SMA0638	*	--	-	-	Hypothetical protein.	1	1
exoD	SMC00353	FlPpPSSyO	--	R	periplasmic	PUTATIVE TRANSMEMBRANE PROTEIN	1	1
fixS2	SMA0622	FnPSO	--	-	periplasmic	FixS2 nitrogen fixation protein.	1	1
ntrR	SMC01521	FlFnPpPSSyO	--	R	-	NITROGEN REGULATORY PROTEIN	1	1
ctaB	SMC00450	CFlFnPpPSSyO	--	H	periplasmic	TRANSMEMBRANE HEME O SYNTHASE	6	15
rkpT2	SMB20822	CPSO	--	-	periplasmic	PUTATIVE CELL SURFACE POLYSACCHARIDE EXPORT ABC-2 TRANSPORTER P	5	5
oppA	SMB21196	CFlFnPpPSSyO	--	-	periplasmic	Oligopeptide ABC transporter (Putative oligopeptide uptake ABC transporter periplasmic solute-binding protein).	1	3
Q92ZC4	SMA1045	*	--	-	periplasmic	Hypothetical protein SMa1045.	1	1
expE8	SMB21307	P	--	-	periplasmic	PUTATIVE PROTEIN	1	1
cgmA	SMC00195	CFnPpPSSyO	--	M	periplasmic	TRANSMEMBRANE CYCLIC BETA-1,2-GLUCAN MODIFICATION PROTEIN	1	3
Q92TC2	SMC02756	CFlFnPpPSSyO	--	T	periplasmic	PUTATIVE SENSOR HISTIDINE KINASE PROTEIN	6	12
exoY	SMB20946	CFlFnPpPSSyO	--	-	periplasmic	Exopolysaccharide production protein exoY.	3	3
nodJ	SMA0863	CFlFnPpPSSyO	--	-	periplasmic	Nodulation protein J	1	2
cyoC	SMB21489	CFlFnPpPSSyO	--	-	periplasmic	PUTATIVE CYTOCHROME O UBIQUINOL OXIDASE CHAIN III (EC1.10.3.-)	4	9
Q92R70	SMC02384	CFlFnPpPSSyO	--	M	periplasmic	PUTATIVE GLYCOSYLTRANSFERASE TRANSMEMBRANE PROTEIN	1	1
ntrC	SMC01043	CFlFnPpPSSyO	--	T	-	NITROGEN ASSIMILATION REGULATORY PROTEIN	4	4
gntK	SMB21119	CFlFnPpPSSyO	--	-	-	Putative gluconokinase protein (EC 2.7.1.12).	1	1
Q92WJ9	SMB20354	Fn	--	-	-	Hypothetical protein SMb20354.	1	1
**syrM**	SMA0849	CFlFnPpPSSy	--	-	periplasmic	HTH-type transcriptional regulator syrM (Symbiotic regulator)	7	7
expE5	SMB21310	*	--	-	-	PUTATIVE MEMBRANE-ANCHORED PROTEIN	2	2
rkpA	SMC02273	CFlFnPpPSSyO	--	Q	-	TRANSMEMBRANE FATTY ACID SYNTHASE	6	16
exoH	SMB20954	Pp	--	-	-	Succinoglycan biosynthesis protein exoH.	1	1
nodN2	SMC03927	CFnPpPSSyO	--	I	-	NODULATION PROTEIN	1	2
expE7	SMB21308	CFlFnPpPSSyO	--	-	periplasmic	Putative glycosyltransferase, forming alpha glycosyl linkages protein.	1	1
expA23	SMB21320	CFlFnPpPSSyO	--	-	periplasmic	PUTATIVE BIFUNCTIONAL GLYCOSYLTRANSFERASE, FORMING BETA-GLYCOS'	4	5
exoL	SMB20956	FnPp	--	-	periplasmic	Succinoglycan biosynthesis protein exoL (EC 2.-.-.-).	2	2
exoP	SMB21506	CFlFnPpPSSyO	--	-	-	Putative auxiliar protein involved in export of cell surface polysaccharides, MPA1 family without cytosolic domain, slightly exc	2	2
Q92SQ4	SMC00393	*	--	R	periplasmic	HYPOTHETICAL/UNKNOWN PROTEIN	1	1
lpsB	SMC01219	CFlFnPpPSSyO	--	M	-	PUTATIVE LIPOPOLYSACCHARIDE CORE BIOSYNTHESIS MANNOSYLTRANSFERASE PROTEIN	1	1
nifA	SMA0815	CFlFnPpPSSyO	--	-	-	Nif-specific regulatory protein.	3	3
Q92NX9	SMC04199	*	--	-	periplasmic	HYPOTHETICAL PROTEIN	1	1
exsF	SMB20934	CFnPpPSO	--	-	periplasmic	ExsF protein (Putative two-component response regulator protein).	1	1
exsC	SMB20939	CFlFnPpPSSyO	--	-	cytoplasmic	ExsC protein (Putative 6-pyruvoyl tetrahydropterin synthase protein) (EC 4.2.3.12).	1	1
fixK	SMA1207	CFlFnPpPSSyO	--	-	-	FixK-like regulatory protein.	1	1
exoI	SMB20951	FnPpPSO	--	-	periplasmic	Succinoglycan biosynthesis protein exoI	8	9
Q92VQ4	SMB21066	CFlFnPpPSSyO	--	-	-	Putative glycosyltransferase protein.	1	1
fixQ	SMA1214	FnP	--	-	periplasmic	FIXQ1 CBB3-TYPE CYTOCHROME OXIDASE	2	2
ctaC	SMC00009	CFlFnPpPSSyO	--	C	periplasmic	CYTOCHROME C OXIDASE SUBUNIT II (EC1.9.3.1)	4	16
nodD1	SMA0870	CFlFnPpPSSyO	--	-	periplasmic	NODD1 TRANSCRIPTION REGULATOR	3	6
exoV	SMB20949	FlPp	--	-	-	Succinoglycan biosynthesis protein exoV.	1	1
expE6	SMB21309	*	--	-	periplasmic	Putative membrane protein.	1	1
rkpZ2	SMB20823	CPO	--	-	-	PUTATIVE CELL SURFACE SACCHARIDE SYNTHESIS PROTEIN, POSSIBLY INVOL	6	6
Q92U35	SMB21481	CFlFnPpPSSyO	--	-	periplasmic	Hypothetical protein SMb21481.	1	1
fdxN	SMA0811	CFlFnPpPSSyO	--	-	periplasmic	Ferredoxin-like protein in nif region.	1	1
expA4	SMB21321	PS	--	-	-	PUTATIVE MEMBRANE-ANCHORED PROTEIN	2	2
bacA	SMB20999	CFlFnPpPSSyO	--	-	periplasmic	Bacteroid development protein bacA	2	8
Q92ZY7	SMA0594	*	--	-	periplasmic	Hypothetical protein.	1	1
Q92MB8	SMC00662	*	--	-	cytoplasmic	HYPOTHETICAL/UNKNOWN PROTEIN	1	1
ivdH	SMB21121	CFlFnPpPSSyO	--	-	periplasmic	Putative isovaleryl-CoA dehydrogenase protein (EC 1.3.99.10).	1	7
fixI2	SMA0621	CFlFnPpPSSyO	--	-	periplasmic	FixI2 E1-E2 type cation ATPase.	1	1
nirK	SMA1250	CFnPpPSSy	--	-	periplasmic	PUTATIVE NIRK CU-NITRITE REDUCTASE (EC1.7.99.3)	1	1
Q930I1	SMA0403	Fn	--	-	periplasmic	Hypothetical protein.	1	1
Q92YN2	SMA1545	CFlFnPpPSSyO	--	-	periplasmic	Putative oxidoreductase (EC 1.6.5.3).	1	1
Q92ZH8	SMA0939	CFlFnPpPSSyO	--	-	periplasmic	Probable sensor histidine kinase of two-component system.	2	6
nifS	SMC00529	CFlFnPpPSSyO	--	E	periplasmic	PYRIDOXAL-PHOSPHATE-DEPENDENT AMINOTRANSFERASE	4	14
expG	SMB21317	CFnPpPS	--	-	-	TRANSCRIPTIONAL ACTIVATOR OF EXOPOLYSACCHARIDE II SYNTHESIS, MARR FAMILY	2	2
Q92T26	SMC02836	CFlFnPpPSSyO	--	V	periplasmic	PUTATIVE ATP-BINDING ABC TRANSPORTER PROTEIN	1	2
Q92VQ5	SMB21065	*	--	-	periplasmic	Hypothetical protein SMb21065.	1	1
ntrP	SMC03949	FnPpS	--	S	cytoplasmic	NITROGEN REGULATORY PROTEIN	1	1
exoT	SMB20950	CFlFnPpPSSyO	--	-	periplasmic	Succinoglycan biosynthesis transport protein exoT	11	15
exsD	SMB20938	CFnPpPSSyO	--	-	-	ExsD protein (Hypothetical conserved protein).	1	1
expD1	SMB21316	CFlFnPpPSSyO	--	-	periplasmic	PUTATIVE ABC TRANSPORTER PROTEIN, FUSED ATP-BINDING AND MEMBRANE	3	31
tufA	SMC01311	CFlFnPpPSSyO	--	--	-	Elongation factor Tu (EF-Tu).	15	15
**dctA**	SMB20611	CFlFnPpPSSyO	--	-	periplasmic	C4-DICARBOXYLATE TRANSPORT PROTEIN	2	2
Q930C1	SMA0525	CFlFnPpPSSyO	--	-	-	Putative ABC-type iron transport system protein.	1	3
rkpG	SMC02272	CFlFnPpPSSyO	--	H	-	ACYL-TRANSFERASE TRANSFERASE	4	8
Q92UC3	SMB20913	*	--	-	periplasmic	Hypothetical protein SMb20913.	1	1
nirV	SMA1247	CFnPpPSO	--	-	periplasmic	periplasmic nitrate reductase (Accessory protein for dissimilatory nitrite reduction)	2	2
acpS	SMC02654	CFnPpPSSyO	--	I	-	PROBABLE HOLO-ACYL-CARRIER PROTEIN SYNTHASE	3	3
norE	SMA1279	CFlFnPpPSSyO	--	-	periplasmic	protein involved in nitric oxide reduction (EC 1.7.99.7)(Cytochrome c oxidase subunit III-like protein)	6	6
exoN2	SMC04023	CFlFnPpPSSyO	--	M	periplasmic	UTP--GLUCOSE-1-PHOSPHATE URIDYLYLTRANSFERASE (EC2.7.7.9)	1	1
noeA	SMA0773	*	--	-	periplasmic	Nodulation protein noeA.	1	1
