# Supplementary material for: The Symbiosis Interactome: a computational approach reveals novel components, functional interactions and modules in Sinorhizobium meliloti
Source: BMC Syst Biol. 2009 Jun 16;3:63. doi: 10.1186/1752-0509-3-63 (PMC2701930; doi:10.1186/1752-0509-3-63)
Supplement: Additional file 3 — List of 440 classical-known and novel proteins, and 1,041 functional interactions predicted to be part of the Symbiosis Interactome network. Proteins are plotted as gene names according to UNIPROT. Scores represent probability of interactions. [file 1752-0509-3-63-S3.pdf]

Protein 1	Protein 2	Score
cyoB	cyoC	0.95
livH	livM	0.94
fixO3	fixN3	0.93
fixN	fixO	0.92
nuoL	nuoM	0.88
nuoN	nuoM	0.88
Q92YV1	Q92YV2	0.83
etfA1	etfB1	0.83
fixA	fixB	0.82
Q92YN6	Q92YN5	0.81
nifD	nifK	0.81
nifE	nifK	0.81
Q92LQ6	Q92LQ7	0.80
fixN	fixO2	0.80
fixN	fixO3	0.80
fixO	fixN3	0.80
fixO2	fixN3	0.80
nuoN	nuoL	0.78
nuoL	Q92YN6	0.77
nuoN	Q92YN6	0.77
Q92MM7	livM	0.76
livK	livM	0.75
hpn8	hpn7	0.75
livJ	livM	0.74
Q92XP8	Q92XP7	0.73
rpsD	rpsH	0.73
Q92YV2	etfB1	0.73
Q92YV1	etfA1	0.73
nodP	nodQ	0.73
nodQ2	nodP2	0.73
rpsG	rpsL	0.73
rpsG	fusA	0.73
rplQ	rpoA	0.73
secY	rpsH	0.73
livM	livG	0.72
Q92X27	Q92X26	0.72
expA8	expA9	0.72
ntrB	ntrC	0.72
cyoA	cyoB	0.72
secY	rpoA	0.72
expA9	expA10	0.72
fabG	fabD	0.72
livM	Q92ZZ6	0.72
expA7	expA8	0.72
rpsH	rpsN	0.72
ctaB	cyoB	0.72
Q92LQ5	Q92LQ4	0.71
fusA	tufA	0.71
fixJ	fixL	0.71
rpsD	rpoA	0.71
Q92LQ6	Q92LQ5	0.71
adk	secY	0.71
Q92UL3	nfeD	0.71
nfeD	Q92UL3	0.71

Q92MM8	Q92MM7	0.71
expD2	expD1	0.71
livH	livK	0.71
livG	livF	0.70
nuoK1	nuoL	0.70
nirD	nirB	0.70
fixO2	fixP2	0.70
nuoJ	nuoK1	0.69
rpsJ	tufA	0.69
ntrX	ntrY	0.69
rpoA	rpsH	0.69
Q92VL7	Q92VL6	0.69
Q92MU6	Q92MU5	0.69
secY	rpsD	0.68
livJ	livH	0.68
dctB	dctD	0.68
Q92TN4	Q92TN3	0.68
Q92MM9	Q92MN0	0.67
fixO	fixP2	0.67
fixB	Q92YV1	0.67
fixB	etfB1	0.67
Q92MM8	Q92MM9	0.67
Q92MM7	livJ	0.66
fixP2	fixO3	0.66
Q92LJ0	Q92LJ1	0.66
Q92MM7	livK	0.66
Q92YF5	Q92YF4	0.65
Q92TN3	Q92LQ6	0.65
Q92ZZ6	Q92MM7	0.64
Q92KZ5	Q92TG8	0.64
rpsE	rpsH	0.63
rpsC	rpsE	0.63
expA7	expA9	0.63
nirB	narB	0.63
rpsJ	rpsC	0.62
rpsC	rpsS	0.62
rpsJ	rpsS	0.62
fusA	rpsL	0.62
nodP	nodQ2	0.62
nodQ	nodP2	0.62
expA8	expA10	0.62
cyoC	cyoA	0.62
livH	livG	0.62
rpsE	secY	0.62
rpsG	tufA	0.62
fabF	fabD	0.62
expA7	expA10	0.62
ctaE	ctaC	0.62
ctaB	ctaC	0.61
livF	livM	0.61
livF	livH	0.61
rpsE	rpsD	0.61
nifE	nifN	0.61
Q92P54	fabD	0.61
Q92TN4	Q92LQ7	0.61

hpn8	Q92XP7	0.61
hpn8	Q92X27	0.61
dctP	Q92WI1	0.61
Q92UM2	dctP	0.61
ctaB	ctaE	0.61
rpsC	secY	0.61
rpsL	tufA	0.61
dctP	Q92WB7	0.61
rpoA	rpsC	0.61
fixO	fixP	0.60
hbdA	Q92KZ5	0.60
rpsS	rpsN	0.60
Q92LQ5	Q92LQ7	0.60
rpsC	rpsG	0.60
acpP	fabF	0.60
ctaB	cyoC	0.60
nifB	nifN	0.60
nifB	nifK	0.60
nuoJ	nuoL	0.60
rpsC	rpsD	0.60
rpoA	rpsE	0.60
nosZ	nosD	0.60
nosY	nosL	0.60
Q92YN2	nuoK2	0.60
exoA	exoM	0.60
norQ	norD	0.60
norC	norB	0.60
fixN	fixP	0.60
fixC	fixX	0.60
rplL	rplJ	0.60
nodB	nodC	0.60
nosZ	nosR	0.60
rpsH	rpsG	0.60
exsC	exsD	0.60
nosD	nosF	0.60
nuoM	nuoJ	0.60
Q92MM7	Q92MM9	0.60
fixB	fixC	0.60
nosY	nosF	0.60
rpsS	rpsE	0.60
rpsG	rpsE	0.60
rpsH	rpsS	0.60
fixP3	fixO3	0.60
rpsN	rpsE	0.60
nifN	nifD	0.60
nirD	narB	0.60
rkpT1	rkpS	0.59
fixG	fixH	0.59
secY	rplQ	0.59
livF	livK	0.59
rpsH	fusA	0.59
rpsE	fusA	0.59
rpsH	rpsL	0.59
livG	livK	0.59
rpsL	rpsJ	0.59

nifH	nifD	0.59
rpsD	rplQ	0.59
Q92LQ8	Q92LQ7	0.59
rpsC	fusA	0.59
norB	norQ	0.59
tyv	Q92WU6	0.59
rpsS	tufA	0.59
exoM	exoO	0.59
nuoJ	nuoN	0.59
exoA	exoL	0.59
rpsC	rpsH	0.59
nuoK1	nuoM	0.59
rpsE	rpsL	0.59
Q92P54	Q92P53	0.59
rpsJ	secY	0.59
dctQ	dctM	0.59
nodE	fabD	0.59
rpsG	rpsJ	0.59
ntrB	nifR33	0.59
fixQ3	fixP3	0.59
rpsH	tufA	0.58
fixB	fixX	0.58
Q92P52	Q92P53	0.58
fusA	rpsJ	0.58
fixN3	fixP3	0.58
Q92MN0	Q92MM7	0.58
rpsJ	rpoA	0.58
rpsS	rpsL	0.58
nuoL	nuoG1	0.58
acpP	nodE	0.58
fusA	rpsS	0.58
tufA	rpsE	0.58
rpsE	rpsJ	0.58
rkpA	rkpG	0.58
Q92WU8	Q92WU7	0.58
Q92WY0	Q92WY1	0.58
Q92WB7	Q92WB8	0.58
nosL	nosX	0.58
Q92NF4	Q92KZ5	0.58
rpmD	rpsE	0.58
rpsG	rpsS	0.58
Q92ZZ6	livH	0.58
dctP	dctM	0.58
Q92U72	Q92U73	0.58
rpsJ	rpsH	0.58
nuoJ	Q92YN6	0.58
fixP	fixO2	0.58
nodA	nodB	0.58
rpsC	rplQ	0.58
fixA	Q92YV2	0.58
ndiA-2	ndiA-1	0.58
ndiB	ndiA-2	0.58
fdxN	nifB	0.58
fixA	etfA1	0.58
exoF	exoQ	0.58

rpsH	rplQ	0.58
rpsC	rpsL	0.58
rpsG	rplQ	0.58
rpsG	rplJ	0.58
rkpZ1	rkpT1	0.58
Q92UM2	Q92UM1	0.57
livH	Q92MM8	0.57
fusA	rplQ	0.57
Q92YL7	Q92YL8	0.57
plsX	fabD	0.57
fixH	fixI	0.57
rpsE	rplQ	0.57
secY	rpsG	0.57
fixG	fixO	0.57
ntrR	ntrP	0.57
fixN	fixP2	0.57
fixP	fixO3	0.57
secY	fusA	0.57
ctaC	cyoB	0.57
tyv	Q92WU4	0.57
Q92MM8	livM	0.57
secY	rpsS	0.57
secY	infA	0.57
Q926F8	Q92UN0	0.57
nolF	nolG	0.57
infA	rpoA	0.57
acpP	Q92P54	0.57
Q92WM5	Q92WM4	0.57
rpsJ	rplQ	0.57
Q92YN6	nuoK1	0.57
rpsJ	nusG	0.57
Q92MN0	Q92MM8	0.57
exoH	exoK	0.57
tufA	rpsC	0.57
Q92YN3	Q92YN5	0.57
rpsS	rpoA	0.57
rpsN	secY	0.57
rplQ	rpsL	0.57
livF	Q92MM7	0.57
etf	Q92YV2	0.57
etfA1	etf	0.57
Q92MM9	livH	0.57
adk	rpsE	0.56
livJ	livF	0.56
rpsN	rpsJ	0.56
fixN3	fixP2	0.56
rpsC	rpsN	0.56
Q92WK0	Q92WJ9	0.56
livJ	livG	0.56
rpsS	rplQ	0.56
fixP	fixN3	0.56
Q92LQ6	livH	0.56
Q92WU9	Q92WU8	0.56
livH	Q92TN3	0.56
rpsG	rpoA	0.56

fixO	fixP3	0.56
nuoG1	nuoJ	0.56
fixP3	fixO2	0.56
fixG	fixO2	0.56
Q92MM7	livG	0.56
Q92VI3	Q92VI0	0.56
fusA	rpoA	0.56
rplQ	rpsN	0.56
Q92V92	Q92V93	0.56
exoN	exoO	0.56
secY	rpsL	0.55
Q92TN3	livM	0.55
fusA	rplJ	0.55
tufA	secY	0.55
fixG	fixO3	0.55
rkpJ	rkpI	0.55
rplJ	nusG	0.55
fixN	fixP3	0.55
Q92WY0	Q92WX9	0.55
Q92XP8	Q92X27	0.55
nusG	tufA	0.55
rpsC	infA	0.55
rplQ	adk	0.55
livM	Q92LQ6	0.55
Q92WI0	Q92WI1	0.55
Q92VU4	Q92VU5	0.55
Q92MM9	livM	0.55
rpsI	rplQ	0.55
rpsH	adk	0.55
Q92PN6	Q92KC1	0.55
Q92XP7	Q92X26	0.55
rpsE	infA	0.55
rpoA	adk	0.55
Q92XP8	hpn7	0.55
greA	lpsB	0.54
Q92X26	hpn7	0.54
noeA	noeB	0.54
tufA	rplQ	0.54
Q92VH5	nodQ2	0.54
acpP	fabD	0.54
exoF	exoY	0.54
Q92WV5	Q92WV3	0.54
Q92ZZ6	livG	0.54
rpsL	rplJ	0.54
livF	Q92ZZ6	0.54
rpsC	adk	0.54
Q92YL8	Q92YL9	0.54
rpoA	rpsL	0.54
tufA	rplJ	0.53
lpsC	lpsD	0.53
Q92R71	Q92R70	0.53
Q930Y4	Q930Y5	0.53
rpsH	infA	0.53
Q92MN0	livH	0.53
Q92LQ5	livF	0.53

fusA2	rpsG	0.53
Q92T68	Q92T65	0.53
acpP	plsX	0.53
adk	rpsS	0.53
fixI2	fixS2	0.53
nuoK2	Q92YN3	0.53
lpsD	lpsE	0.53
Q930I2	Q930I1	0.53
livH	Q92TN4	0.52
livJ	Q92MM8	0.52
expE6	expE7	0.52
Q92YU7	hbdA	0.52
exoK	exoL	0.52
Q92KZ6	Q92KZ5	0.52
rpsJ	adk	0.52
Q92YF3	Q92YF5	0.52
Q92VI0	Q92VI1	0.52
Q92WB6	Q92WB7	0.52
nuoM	Q92YN5	0.52
Q92PN6	dctP	0.52
Q92MM7	Q92TN3	0.52
infA	adk	0.52
Q92TN4	livM	0.52
Q92U73	dctP	0.51
Q92LQ6	Q92MM7	0.51
livH	Q92LQ4	0.51
Q930B1	Q930B0	0.51
Q92YU8	Q92YU7	0.51
Q92Z93	Q92Z92	0.51
Q930V8	Q930V7	0.51
Q930L4	Q930L5	0.51
expE4	expE3	0.51
Q930K4	Q930K3	0.51
Q92Z94	Q92Z93	0.51
Q92ZD6	Q92ZD7	0.51
TRm30.2	TRm30.3	0.51
Q92ZW2	Q92ZW3	0.51
Q92ZW6	Q92ZW7	0.51
Q92ZA5	PHK2_RHI	0.51
Q92ZA0	Q92ZA1	0.51
Q92U46	Q92U45	0.51
Q930B0	Q930A9	0.51
Q92ZA2	Q92ZA1	0.51
Q92N90	Q92N89	0.51
Q92XP6	Q92XP7	0.51
Q92XP8	Q92XP9	0.51
Q92SQ5	Q92SQ4	0.51
Q92U36	Q92U35	0.51
Q92JU8	Q92JU7	0.51
Q92NX9	Q92NX8	0.51
Q92JY8	Q92MB8	0.51
Q92YE7	Q92YE6	0.51
Q92PT0	xthA1	0.51
Q92YE4	Q92YE5	0.51
fixI	fixS	0.51

Q92ZC4	Q92ZC3	0.51
oppA	Q92V56	0.51
Q92ZY7	Q92ZY8	0.51
Q92XM4	Q92XM5	0.51
Q92PQ3	Q92PQ4	0.51
Q92XM0	Q92XL9	0.51
Q92V38	pphA	0.51
Q92UC3	Q92UC2	0.51
Q92ZU2	Q92ZU3	0.51
Q92WP1	Q92WP2	0.51
Q92VQ5	Q92VQ4	0.51
Q92VK2	gntK	0.51
expA1	expA23	0.51
ivdH	Q92VK2	0.51
expA5	expA6	0.51
gst9	Q92T68	0.51
Q92VQ6	Q92VQ7	0.51
Q930C1	Q930C2	0.51
expE3	expE2	0.51
Q92UD1	Q92UD2	0.51
exsF	exsG	0.51
Q92LQ5	livM	0.51
Q92LQ5	livG	0.51
dctP	Q92WP2	0.51
rpsS	rpsD	0.51
dctP	Q92WM5	0.51
Q92MM8	livK	0.51
nuoN	Q92YN5	0.51
exoN	exoP	0.50
hbdA	Q92NF4	0.50
nusG	rpsC	0.50
rpsJ	rpsD	0.50
nodN	nolG	0.50
livM	Q92MN0	0.50
Q930L3	Q930L5	0.50
Q92MN0	livF	0.50
nifH	nifK	0.50
rplJ	rpsJ	0.50
livH	Q92LQ7	0.50
nosY	nosD	0.49
nosL	nosD	0.49
nosZ	nosY	0.49
nosD	nosR	0.49
norC	norQ	0.49
exoB	galM	0.49
nosZ	nosL	0.49
Q92MM9	livJ	0.49
nuoK1	nuoN	0.49
norB	norD	0.49
nosY	nosR	0.49
Q92LQ4	Q92LQ6	0.49
nosL	nosR	0.49
Q92LQ5	Q92MM8	0.49
fusA2	rpsL	0.49
fixA	fixC	0.49

norC	norD	0.49
nosL	nosF	0.49
Q92LQ7	Q92LQ4	0.49
cyoA	ctaB	0.49
nosZ	nosF	0.49
paaG	hbdA	0.49
nifE	nifH	0.49
Q92ZZ6	Q92MM8	0.49
fixG	fixP	0.48
fixA	fixX	0.48
exoA	exoO	0.48
nosF	nosR	0.48
Q92MN0	livG	0.48
exoL	exoM	0.48
ntrC	nifR33	0.48
fixG	fixN	0.48
rpsG	rpsI	0.48
nodE	nodG	0.48
fixG	fixI	0.48
tufA	fusA2	0.48
ntrC	ntrX	0.48
Q926F8	Q92WV5	0.48
rkpZ1	rkpS	0.48
acpP	fabB	0.48
nosY	nosX	0.48
nifN	nifX	0.47
Q92T65	Q926F8	0.47
Q92YV1	etf	0.47
etfB1	etf	0.47
Q92QS4	Q92QS6	0.47
Q92TN4	Q92MM7	0.47
nodA	nodC	0.47
Q92MU6	lacK1	0.47
aglK	Q92MU6	0.47
exoL	exoO	0.47
fixG	fixP2	0.47
rpsC	rplJ	0.47
nifH	nifN	0.47
Q92YU7	Q92KZ5	0.47
Q92UQ2	Q926F8	0.47
nifB	nifD	0.47
Q92VL6	Q92TH4	0.47
Q92LJ1	Q92MU6	0.47
Q92YF4	Q92YF3	0.47
dctP	dctQ	0.47
rkpG	rkpH	0.47
Q92WV3	Q92MU6	0.47
Q92PP2	dctP	0.47
nifB	nifE	0.46
rkpS	rkpR/kpsE	0.46
rpsI	rpsE	0.46
nosD	nosX	0.46
rplJ	rpsS	0.46
Q92P67	Q92TH4	0.46
nifB	nifH	0.46

nusG	secY	0.46
exoM	exoP	0.46
Q92TX6	Q92MU6	0.46
fixG	fixN3	0.46
Q926F8	Q92YW8	0.46
Q92LQ7	livM	0.46
Q92TX6	Q92WV5	0.46
Q92TN3	livJ	0.46
Q92TH4	Q92TX6	0.46
Q92WV5	Q92WV7	0.46
Q92TH4	Q92VI3	0.46
nifE	nifD	0.46
Q930K4	Q930K5	0.46
rpsD	rpsL	0.46
rpsI	rpsH	0.46
rpsD	fusA	0.46
dctA	dctB	0.46
fixG	fixP3	0.46
livK	Q92MM9	0.46
Q92TX6	Q92XK1	0.45
nosF	nosX	0.45
rpsG	rpsD	0.45
exoA	exoP	0.45
Q92TX6	Q92YW8	0.45
Q92LQ5	Q92MM9	0.45
Q92LJ1	Q92TH4	0.45
exoB	galE	0.45
syrA	syrM	0.45
fixJ	nifA	0.45
Q926F8	Q92LJ0	0.45
Q92XK1	Q92VI3	0.45
Q92LQ7	Q92MM7	0.45
Q92WU4	rmlB	0.45
nosZ	nosX	0.45
Q92XK1	Q92P67	0.45
Q92MU5	Q92WV5	0.45
fixG	fixS	0.45
ctaE	cyoB	0.45
Q92TH4	Q92WV3	0.45
exoA	exoK	0.45
rkpS	rkpT2	0.45
rkpJ	rkpZ2	0.45
Q92VI3	Q92YW8	0.44
Q92TX6	Q92UN0	0.44
Q92ZS2	Q92TX6	0.44
exoO	exoP	0.44
livK	Q92TN3	0.44
Q92YW8	lacK1	0.44
rkpJ	rkpZ1	0.44
exoV	exoW	0.44
lpsC	lpsE	0.44
efp	rpsD	0.44
nusG	rho	0.44
Q92LJ0	Q92TX6	0.44
Q92LJ1	Q92ZS2	0.44

chvG	Q92TC2	0.44
Q92ZZ6	Q92MM9	0.44
livJ	Q92LQ6	0.44
Q92VI0	Q92VL6	0.44
exoL	exoP	0.44
acpP	nodG	0.44
Q92VI3	Q92WV5	0.44
exoM	exoN	0.43
Q92YX9	Q92TX6	0.43
Q92TX8	Q92WV3	0.43
Q92WV5	Q92LJ1	0.43
Q92WV3	Q92YW8	0.43
Q92YW8	Q92VL6	0.43
Q92LJ1	Q92YW8	0.43
Q92YX9	Q92WV3	0.43
Q92VL7	lacK1	0.43
Q92VH5	nodP2	0.43
thuK	Q92ZS2	0.43
rpsE	rplJ	0.43
Q92ZS2	Q92MU5	0.43
fixK	fixT	0.43
Q92ZS2	Q92P67	0.43
rpsS	rpsI	0.43
Q92ZS2	lacK1	0.43
Q92P67	Q92YW8	0.43
Q92T68	Q92YW8	0.43
Q92VL7	Q92VI3	0.43
fixN2	fixO2	0.43
Q92T68	Q92ZS2	0.43
Q92VL7	Q92TX6	0.43
rkpH	rkpI	0.43
rpsH	rplJ	0.43
Q92P67	Q92WV5	0.43
Q92LQ6	livK	0.43
exoK	exoM	0.43
Q92VI3	Q92UN0	0.43
exoU	exoW	0.42
Q92XP8	hpn8	0.42
rkpR/kpsE	rkpT2	0.42
rkpT1	rkpR/kpsE	0.42
aglK	Q92ZS2	0.42
rkpJ	rkpR/kpsE	0.42
nusG	rpoA	0.42
exoA	exoN	0.42
Q92YW8	thuK	0.42
exoA	exoH	0.42
exoH	exoL	0.42
fixH	fixS	0.42
Q92XK1	Q92WV3	0.42
Q92LJ1	Q92U55	0.42
Q92XK1	Q92LJ1	0.42
thuK	Q92WV5	0.42
Q92U55	Q92WV3	0.42
exoL	exoN	0.42
Q92WV5	Q92T68	0.42

engA	rpsH	0.42
rkpJ	rkpS	0.41
aglK	Q92VI0	0.41
Q92XK1	Q92VL6	0.41
Q92LJ0	Q92MU5	0.41
Q92VL7	Q92WV3	0.41
rpsI	rpsC	0.41
Q92ZS2	Q92VL6	0.41
Q92VL7	Q92LJ1	0.41
Q92VL7	Q92MU5	0.41
Q92TX8	Q92TX6	0.41
Q92VL6	Q92YX9	0.41
Q92WV3	Q92UN0	0.41
rpsI	rpsD	0.41
nifN	nifK	0.41
livJ	Q92TN4	0.41
livG	Q92LQ6	0.41
exoQ	exoY	0.41
htpG	adk	0.41
Q92LQ4	livF	0.40
Q92VI0	Q92WV3	0.40
rpsH	nusG	0.40
Q92LJ1	Q92VI0	0.40
Q92NC7	hbdA	0.40
exoK	exoO	0.40
Q92VL6	Q92UN0	0.40
Q92TN3	Q92ZZ6	0.40
secY	rplJ	0.40
rplJ	rplQ	0.40
Q92MU5	Q92T65	0.40
Q92ZS2	Q92WV3	0.40
Q92LQ6	Q92ZZ6	0.40
Q92TN3	Q92LQ5	0.40
hpn8	Q92X26	0.40
rkpZ1	rkpR/kpsE	0.39
rpsJ	infA	0.39
expE4	expE2	0.39
exoK	exsH	0.39
nifB	nifX	0.39
Q92YX9	lacK1	0.39
Q92YX9	Q92LJ1	0.39
lacK1	Q92LJ0	0.39
dctP	Q930W3	0.39
exoH	exoM	0.39
Q92P67	Q92LJ0	0.39
rkpR/kpsE	rkpZ2	0.39
Q92LQ6	Q92MN0	0.39
Q92YU7	Q92YV2	0.39
Q92MU5	Q92YX9	0.39
lacK1	Q92T65	0.39
Q92T65	Q92TX6	0.39
expE4	expE7	0.38
Q92LQ5	livK	0.38
Q930Y5	Q92R78	0.38
rkpS	rkpZ2	0.38

Q92LQ4	Q92TN3	0.38
exoA	exoU	0.38
lacG	Q92MU5	0.38
Q92YX9	Q92VI3	0.38
Q92LJ0	Q92T68	0.38
Q92LQ5	Q92TN4	0.38
exoM	exoQ	0.38
Q92LJ0	Q92WV3	0.38
Q92PN6	dctQ	0.38
Q92LQ4	livG	0.38
rkpJ	rkpT2	0.38
expA5	expA7	0.38
livG	Q92MM8	0.38
norC	nosR	0.38
nodN	nolF	0.38
rkpZ1	rkpT2	0.37
Q92LQ4	Q92TN4	0.37
Q92UM1	dctP	0.37
fixO	fixS2	0.37
Q92T65	Q92LJ1	0.37
dctQ	Q92WB7	0.37
rkpJ	rkpT1	0.37
rkpZ2	rkpT2	0.37
exoM	exoU	0.37
thuK	Q92YX9	0.36
Q92UM1	dctM	0.36
expE1	expD1	0.36
Q92U73	dctQ	0.36
Q92UN0	Q92WV7	0.36
Q92LQ5	Q92ZZ6	0.36
exoK	exoN	0.36
Q92MU5	Q92TH4	0.36
Q92U72	dctM	0.36
hupB	ihfB	0.36
rmlB	tyv	0.36
dctQ	Q92WI1	0.36
nifA	nifB	0.36
exoO	exoT	0.36
hbdA	Q92TG8	0.35
nosZ	norB	0.35
aglK	Q92TH4	0.35
expE1	expD2	0.35
exoI	exoT	0.35
lacK1	Q92TH4	0.35
Q92MM8	Q92LQ4	0.35
Q92YW8	Q92WV7	0.35
Q92YX9	Q92P67	0.35
aglK	Q92T65	0.35
Q92UQ2	Q92WV3	0.34
exoP	exoT	0.34
Q92YX9	Q92T68	0.34
exoL	exoT	0.34
dctQ	Q92WM5	0.34
fdxN	fixX	0.34
rkpT1	rkpZ2	0.34

Q92UM2	dctQ	0.34
livJ	Q92LQ7	0.34
dctQ	Q92WP2	0.34
Q92YW8	Q92MU5	0.34
Q92TH4	Q92T68	0.34
expA5	expA9	0.34
Q92WV5	lacK1	0.34
chvG	exoR	0.34
nosZ	norC	0.34
norB	nosR	0.34
ctaC	norE	0.34
Q92TN3	Q92MN0	0.33
Q92XK1	lacK1	0.33
Q92MU5	Q92XK1	0.33
cyoB	norE	0.33
rpsD	engA	0.33
ndvA	Q92T26	0.33
exoM	exoT	0.33
livK	Q92LQ7	0.33
exoP	exoQ	0.33
Q92TX6	Q92VI0	0.33
exoK	exoP	0.33
fixN2	fixO3	0.33
expE2	expE7	0.32
exoH	exoN	0.32
fixO	fixN2	0.32
Q92P67	Q92UN0	0.32
Q92XK1	aglK	0.32
Q92LQ7	Q92MN0	0.32
aglK	Q92YX9	0.32
thuK	Q92XK1	0.32
fixK	fixN	0.32
nodG	fabG	0.32
exoA	exoT	0.32
exoH	exoP	0.32
aglK	Q92WV5	0.32
Q92VL6	Q92WV5	0.32
aglK	Q92YW8	0.31
Q92NC7	Q92YU7	0.31
Q92UQ2	Q92MU5	0.31
Q92UN0	Q92LJ1	0.31
rkpH	rkpJ	0.31
Q92UQ2	Q92TX6	0.31
Q92TG8	Q92NF4	0.31
exoF	exoP	0.31
rkpG	rkpJ	0.31
exoU	exoV	0.30
dctM	Q930W3	0.30
expG	expC	0.30
Q92ZS2	Q926F8	0.30
dctQ	Q930W3	0.30
expE4	expA23	0.30
ntrC	ntrY	0.30
Q930W3	Q92PN6	0.30
ctaE	norE	0.30

exoP	exoY	0.30
acpP	rkpA	0.30
ndvA	Q92MM9	0.30
nodD1	nodD2	0.30
acpP	fabG	0.30
exoQ	exoT	0.30
syrM	nodD1	0.30
exoA	exoV	0.30
ntrY	Q92TC2	0.30
exoO	exoW	0.30
acpS	lepB	0.30
exoB	exoF	0.30
exsA	Q92LQ7	0.30
exoM	exoV	0.30
Q92MN0	livJ	0.30
dctM	Q92WP2	0.30
exoF	exoH	0.30
nodE	nodF	0.30
ntrB	Q92ZH8	0.30
ntrB	Q92YF5	0.30
nodE	phbB	0.30
exoI	exoO	0.30
exoA	exoB	0.30
nosD	norB	0.30
nosL	norB	0.30
exoT	exoW	0.30
rkpG	rkpI	0.30
livK	Q92MN0	0.30
hupB	greA	0.30
bacA	exsA	0.30
ndvA	Q92VI3	0.30
exoF	exoO	0.30
exsA	Q92TN3	0.30
Q92YU7	Q92TG8	0.30
expA10	tyv	0.30
exoA	exoW	0.30
exoT	exoV	0.30
Q92YN5	nuoL	0.30
nodI	nodJ	0.30
exoO	exoU	0.30
nifN	nifS	0.30
fixJ	fixT	0.30
rpsG	engA	0.30
kpsF3	kpsF1	0.30
fixN	fixN3	0.30
adk	nifS	0.30
nodE	Q92P52	0.30
dctM	Q92WB8	0.30
fixN2	fixG	0.30
exoL	exoU	0.30
nodE	Q92P54	0.30
Q92YW8	Q92UQ2	0.30
fixN2	fixP2	0.30
ndvA	Q92P67	0.30
expA7	tyv	0.30

expA23	expE7	0.30
norE	cyoC	0.30
ndvA	msbA1	0.30
fixN	fixN2	0.30
Q92YW8	Q92XK1	0.30
fixK	fixL	0.30
etfA1	Q92YU7	0.30
fabF	nodG	0.30
nodG	Q92WB6	0.30
greA	lpsE	0.30
exoF	exoX	0.30
aglK	ndvA	0.30
efp	engA	0.30
Q92UQ2	Q92LJ1	0.30
fixA	nifH	0.30
ndvA	nodI	0.30
exoR	ropB2	0.30
exoL	exoW	0.30
nifH	ntrC	0.30
dctD	nifA	0.30
nirK	nirV	0.30
expE2	Q92VQ4	0.30
fixO	fixO2	0.30
bacA	msbA1	0.30
Q926F8	Q92V93	0.30
exoY	Q92VP9	0.30
nodD2	syrM	0.30
nodG	fabD	0.30
tufA	nodQ	0.30
fixP	fixQ	0.30
exoF	exoT	0.30
Q930W3	Q92UM2	0.30
Q92WM5	Q930W3	0.30
nosY	norC	0.30
exoX	mucR	0.30
dctM	Q92WM5	0.30
chvG	fixJ	0.30
nodQ	nodQ2	0.30
kpsF2	kpsF1	0.30
Q92UM2	dctM	0.30
exoH	exoZ	0.30
rkpA	fabD	0.30
greA	lpsC	0.30
expG	mucR	0.30
fixO	fixO3	0.30
engA	secY	0.30
exoI	exoM	0.30
nosZ	norD	0.30
chvG	ntrB	0.30
expA5	expA4	0.30
nodD3	syrM	0.30
exoL	exoY	0.30
exsA	Q92TN4	0.30
syrM	fixG	0.30
fixJ	fixK	0.30

exoL	exoQ	0.30
ndvA	Q92MU5	0.30
nodG	Q930L5	0.30
exoF	exoL	0.30
exoL	exoV	0.30
nodI	Q92T26	0.30
hupB	nodM	0.30
Q92YW8	Q92UN0	0.30
Q92PN6	dctM	0.30
syrM	exoX	0.30
aglK	Q92LJ0	0.30
acpP	phbB	0.30
ctaB	norE	0.30
Q92WP2	Q930W3	0.30
ntrX	rpoN	0.30
exoI	exoL	0.30
Q92LJ0	Q92YW8	0.30
nodG	phbB	0.30
nodP	nodP2	0.30
engA	infA	0.30
ndvA	Q92MM8	0.30
Q92WI1	Q930W3	0.30
nosL	norC	0.30
phbB	rkpA	0.30
exoX	exoY	0.30
kpsF2	rpoN	0.30
livF	rpoN	0.30
expE8	expE7	0.30
exoB	exoH	0.30
expA7	rmlB	0.30
nifE	nifS	0.30
nodC	nodI	0.30
nodE	plsX	0.30
exoP	exoV	0.30
exoO	exoV	0.30
ndvA	exsA	0.30
Q92Z75	fixL	0.30
dctD	ntrC	0.30
dctD	fixL	0.30
exoM	exoY	0.30
nodP	nodG	0.30
nifA	rpoN	0.30
exoN	exoN2	0.30
nodD1	nodD3	0.30
Q92WB7	Q930W3	0.30
exoA	exoI	0.30
dctM	Q92WB7	0.30
Q92WI1	Q92PP2	0.30
cgmA	rkpI	0.30
fixA	Q92YV1	0.30
fixJ	Q92TC2	0.30
acpP	rplL	0.30
expA8	tyv	0.30
ihfA	hupB	0.30
exoI	exoW	0.30

fixB	etf	0.30
fixB	etfA1	0.30
fixB	Q92YV2	0.30
kpsF1	rpoN	0.30
mocE	mocD	0.30
napA	nirB	0.30
Q92MM9	rpoN	0.30
fixO	fixQ	0.30
Q92PP2	Q92WB7	0.30
Q92LQ7	rpoN	0.30
Q92U72	dctP	0.30
tufA	nodQ2	0.30
etfA1	Q92NF4	0.30
fixA	etfB1	0.30
nifA	nifH	0.30
mocF	mocE	0.30
exoD	syrM	0.30
napA	nifS	0.30
rlpA	nolG	0.30
ntrX	Q92TC2	0.30
exoM	exoW	0.30
nodE	fabG	0.30
nifA	ntrC	0.30
nifA	ntrX	0.30
nodA	nodH	0.30
fixP3	fixN2	0.30
expA4	expA23	0.30
fixL	nifA	0.30
exoA	exoQ	0.30
fixP	fixN2	0.30
Q92YW8	Q92TH4	0.30
kpsF3	kpsF2	0.30
kpsF3	rpoN	0.30
Q92YW8	lacG	0.30
Q92PP2	dctQ	0.30
fabF	nodE	0.30
nodG	plsX	0.30
expE5	expE4	0.30
nodI	expD1	0.30
nosD	norC	0.30
exoI	exoV	0.30
fixA	nifA	0.30
exoF	exoU	0.30
Q92MM7	thuK	0.30
rkpH	fabD	0.30
exoF	ndvA	0.30
ntrB	ntrX	0.30
Q92V93	Q92YW8	0.30
nodN	nodN2	0.30
Q92U73	dctM	0.30
dctA	ntrC	0.30
dctB	fixJ	0.30
Q92NF4	Q92YV2	0.30
cysG	nirB	0.30
Q92MN0	Q92ZZ6	0.30

fixL	Q92ZH8	0.30
exoT	exoU	0.30
fixJ	Q92YF5	0.30
gst9	mocD	0.30
ntrB	Q92TC2	0.30
efp	infA	0.30
rkpA	fabG	0.30
Q930W3	Q92U73	0.30
exoA	exoF	0.30
dctD	Q92TC2	0.30
exoI	exoU	0.30
norD	nirV	0.30
expE5	expE6	0.30
Q92MM8	rpoN	0.30
dctD	ntrX	0.30
nodE	fabB	0.30
chvG	ntrY	0.30
Q930W3	Q92PP2	0.30
chvG	mucR	0.30
fixB	Q92YU7	0.30
fixB	Q92TQ5	0.30
fixJ	Q92ZH8	0.30
nodD2	nodD3	0.30
Q92YW8	Q92ZS2	0.30
engA	rpoA	0.30
expA9	galM	0.30
fixB	Q92NF4	0.30
fixI	fixI2	0.30
rkpZ1	rkpZ2	0.30
fixL	Q92YF5	0.30
nodG	Q92P54	0.30
exoQ	exoV	0.30
Q92QS6	Q92VP9	0.30
ndvA	lacK1	0.30
ndvA	Q92TN3	0.30
Q92WV5	Q92YW8	0.30
dctM	Q92WI1	0.30
nodG	rkpA	0.30
nodG	fabB	0.30
exoQ	exoZ	0.30
exoF	exoM	0.30
greA	lpsD	0.30
nodM	nolF	0.30
expA7	expA6	0.30
Q92UM2	Q92PP2	0.30
efp	rpsG	0.30
exoY	Q92QS6	0.30
dctD	rpoN	0.30
napA	napD	0.30
Q92PP2	dctM	0.30
bacA	Q92T26	0.30
Q92ZZ6	Q92LQ7	0.30
